# COVID-19 relapse associated with SARS-CoV-2 evasion from CD4^+^ T-cell recognition in an agammaglobulinemia patient

**DOI:** 10.1016/j.isci.2023.106685

**Published:** 2023-04-20

**Authors:** Ryo Morita, Ritsuko Kubota-Koketsu, Xiuyuan Lu, Tadahiro Sasaki, Emi E. Nakayama, Yu-chen Liu, Daisuke Okuzaki, Daisuke Motooka, James Badger Wing, Yasunori Fujikawa, Yuji Ichida, Kiyoko Amo, Tetsushi Goto, Junichi Hara, Michinori Shirano, Sho Yamasaki, Tatsuo Shioda

**Affiliations:** 1 Department of Infectious Diseases, Osaka City General Hospital, Osaka 534-0021, Japan; 2 Department of Viral Infections, Research Institute for Microbial Diseases, Osaka University, Osaka 565-0871, Japan; 3 Laboratory of Molecular Immunology, Immunology Frontier Research Center, Osaka University, Osaka 565-0871, Japan; 4 Laboratory of Human Immunology (Single Cell Genomics), Immunology Frontier Research Center, Osaka University, Osaka 565-0871, Japan; 5 Department of Infection Metagenomics, Genome Information Research Center, Research Institute for Microbial Diseases, Osaka University, Osaka 565-0871, Japan; 6 Department of Medical Laboratory, Osaka City General Hospital, Osaka 534-0021, Japan; 7 Laboratory of Human Immunology (Single Cell Immunology), Immunology Frontier Research Center, Osaka University, Osaka 565-0871, Japan; 8 Department of Pharmacy, Osaka City General Hospital, Osaka 534-0021, Japan; 9 Department of Pediatric Emergency Medicine, Osaka City General Hospital, Osaka 534-0021, Japan; 10 Department of Pediatric Hematology and Oncology, Osaka City General Hospital, Osaka 534-0021, Japan

**Keywords:** Immunology

## Abstract

A 25-year-old patient with a primary immunodeficiency lacking immunoglobulin production experienced a relapse after a 239-day period of persistent severe acute respiratory syndrome coronavirus 2 (SARS-CoV-2) infection. Viral genetic sequencing demonstrated that SARS-CoV-2 had evolved during the infection period, with at least five mutations associated with host cellular immune recognition. Among them, the T32I mutation in ORF3a was found to evade recognition by CD4^+^ T cells. The virus found after relapse showed an increased proliferative capacity *in vitro*. SARS-CoV-2 may have evolved to evade recognition by CD4^+^ T cells and increased in its proliferative capacity during the persistent infection, likely leading to relapse. These mutations may further affect viral clearance in hosts with similar types of human leukocyte antigens. The early elimination of SARS-CoV-2 in immunocompromised patients is therefore important not only to improve the condition of patients but also to prevent the emergence of mutants that threaten public health.

## Highlights

• SARS-CoV-2 persistently infected a man with humoral immunodeficiency for 239 days
• COVID-19 relapsed and SARS-CoV-2 evolved during the infection period
• The virus isolated after relapse had an increased proliferative capacity *in vitro*
• SARS-CoV-2 may have evolved to evade recognition by CD4^+^ T-cells

## Graphical abstract

**Figure:**
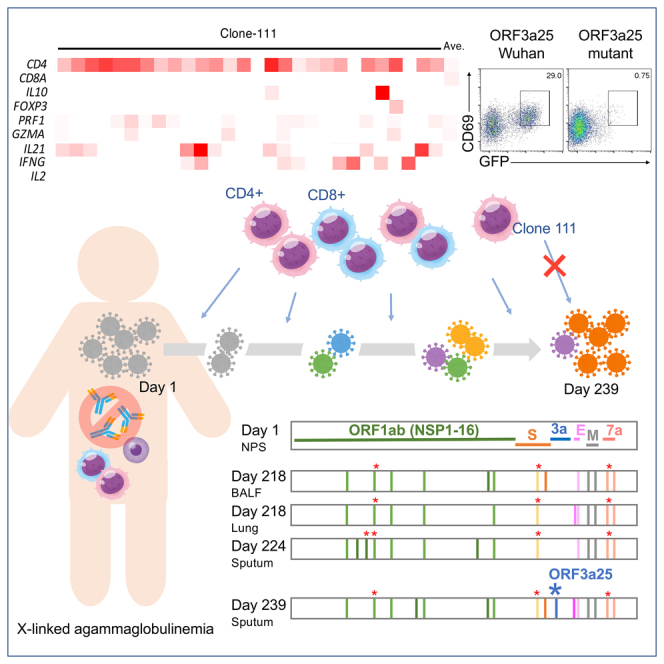

## Introduction

X-linked agammaglobulinemia (XLA) is a rare primary humoral immunodeficiency that was first reported by Bruton in 1952. In XLA, abnormalities in Bruton’s tyrosine kinase prevent normal B-cell maturation, resulting in agammaglobulinemia.^1^ The prognosis is generally good if globulin preparations are administered regularly.^1^

Coronavirus disease 2019 (COVID-19) can become chronic and relapse in patients with primary^2^ and secondary^3^^,^^4^^,^^5^^,^^6^^,^^7^ antibody deficiencies. Previous reports have shown that convalescent plasma,^7^^,^^8^ remdesivir plus convalescent plasma,^5^^,^^9^^,^^10^ and remdesivir plus a monoclonal antibody cocktail^11^ have therapeutic effects in COVID-19 cases with XLA. Polyfunctional CD8^+^ T-cell responses have also been observed during the course of severe acute respiratory syndrome coronavirus 2 (SARS-CoV-2) infection in patients with XLA.^6^ A recent analysis of SARS-CoV-2 infection in an immunocompromised patient demonstrated viral evolution and reduced sensitivity to neutralizing antibodies, which was either dependent on^12^ or independent of convalescent plasma therapy.^13^

Here, we present an XLA case in which SARS-CoV-2 infection persisted for more than 239 days without specific humoral immunity to SARS-CoV-2. The patient experienced a relapse as SARS-CoV-2 evolved during the infection period. The clinical course of the patient, intra-host evolution of SARS-CoV-2 during persistent and relapsing infection for more than 239 days, and SARS-CoV-2-specific T-cell responses in the absence of humoral immunity have been described.

## Results

### Clinical presentation of an agammaglobulinemia patient who experienced relapse following persistent SARS-CoV-2 infection

**Figure 1:**
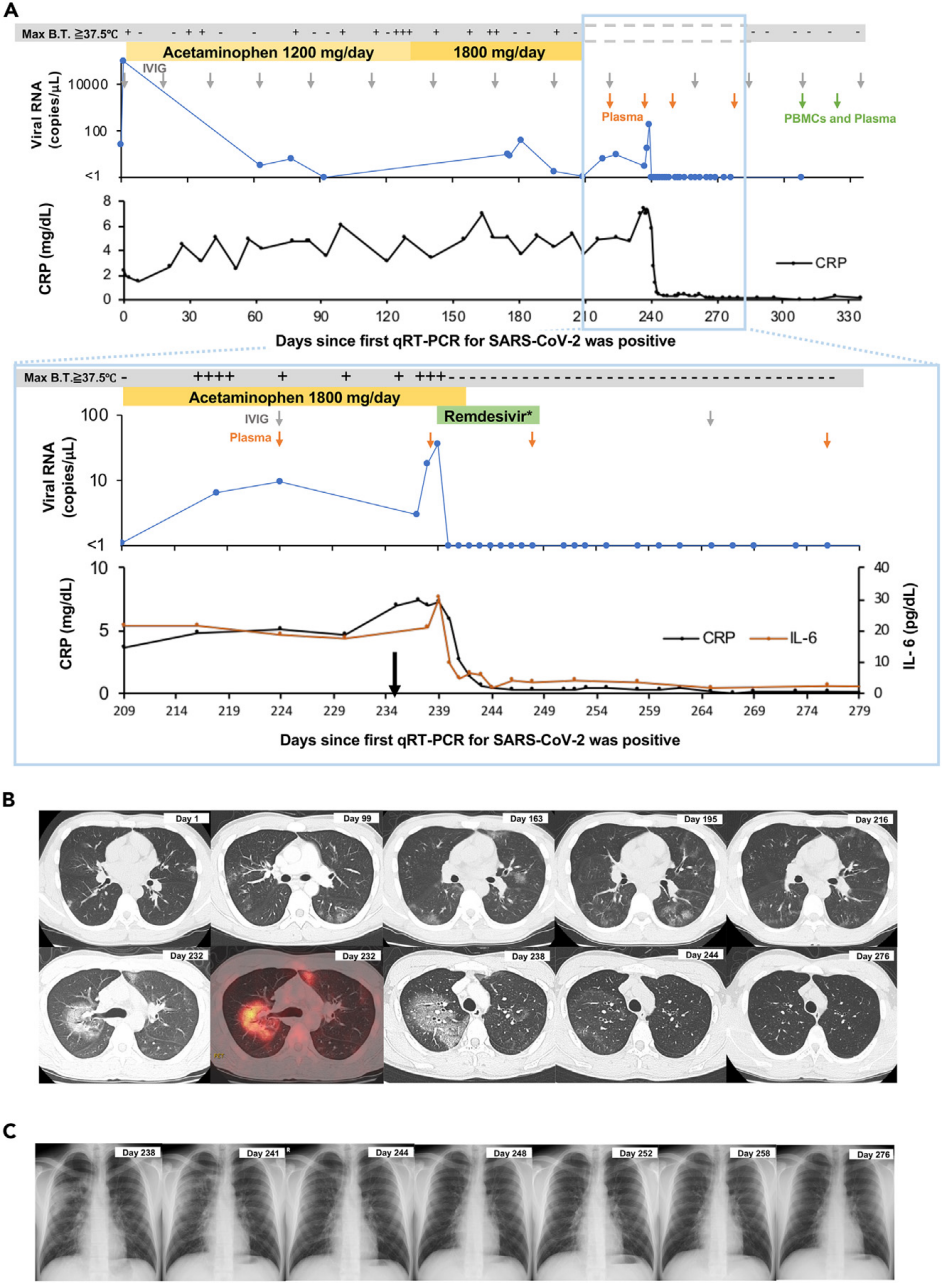
Clinical and laboratory data from the patient during the observation period (A) Results of qRT-PCR for SARS-CoV-2; days in which there was a maximum body temperature above 37.5°C; and blood tests during the observation period. The black arrow indicates the time of symptom exacerbation. The gray arrow indicates the administration time of intravenous immunoglobulin (IVIG). The green and orange arrows indicate the collection time of PBMCs for the T-cell analysis and/or plasma for the measurement of the anti-SARS-CoV-2 spike antibody, respectively. qRT-PCR, real-time quantitative reverse transcriptase polymerase chain reaction; Max B.T., maximum body temperature of the day. ∗ Remdesivir was administered at 200 mg/day on the first day and 100 mg/day for the following 9 days. (B) Computed tomography (black and white images) and positron emission tomography/computed tomography (colored image) results during the observation period. (C) Chest X-ray results during the observation period, particularly after starting the administration of remdesivir. See also Tables S1–S3 and S5.

A 25-year-old male patient developed fever at the end of July 2020. He had been genetically diagnosed with XLA due to an exon 6 skip mutation (splicing site mutation, c.392-2A>G) at 11 months of age. The CD19 lymphocyte subset in this patient was undetectable (Table S1). The same exon 6 skip mutation has been reported to notably reduce the levels of BTK expression in monocytes.^14^ The neutrophil sterilizing function and natural killer cell activity in the patient were within normal limits, but there was a decrease in his neutrophil phagocytosis function (Table S1). He was diagnosed with COVID-19 based on a positive result from a quantitative real-time reverse transcription polymerase chain reaction (qRT-PCR) test for SARS-CoV-2 in early August. One day after the COVID-19 diagnosis (day 1), the patient was admitted to Osaka City General Hospital as this was a requirement from the Japanese government at the time. Because the early prognosis of the patient was thought to be good and his general condition remained stable after he had been admitted, no antiviral drugs, convalescent plasma, or antibody cocktails were administered, and he was discharged on day 9. After being discharged, the patient occasionally experienced malaise and fever. Blood tests showed slightly but persistently elevated levels of C-reactive protein (CRP) (range, 2.51–7.49 mg/dL; median, 4.85 mg/dL) (Figure 1A). Computed tomography (CT) performed on days 99, 163, 195, 216, and 232 showed pneumonia with migrating ground glass opacities, and [18F]-2-fluoro-2-deoxy-D-glucose (FDG)-positron emission tomography (PET)/CT on day 232 showed an accumulation of FDG only at the site of pneumonia (maximum standardized uptake value, 5.6) (Figure 1B). Considering the possibility of organizing pneumonia, a bronchoscopy with bronchoalveolarlavage was performed on day 218, which confirmed non-specific inflammation in the biopsy tissue of the pneumonia-affected region. The qRT-PCR testing for SARS-CoV-2 in sputum and saliva samples taken on days 63, 77, 175, 176, and 181 detected SARS-CoV-2 RNA with high cycle threshold (Ct) values (33.82–36.5; median, 35.3) (Figure 1A). Because the Ct values were high, COVID-19 was initially considered unlikely to be the main cause of the pneumonia and fever.

On approximately day 235, the general condition of the patient worsened significantly. He presented chills, shivering, malaise, and uncontrollably high fever (approximately 40 °C, treated with 1,800 mg of acetaminophen per day). Blood testing showed elevated levels of CRP and interleukin (IL)-6, and CT showed worsening pneumonia. The qRT-PCR test for SARS-CoV-2 on day 239 showed a decreased Ct value. The multiplex PCR testing of sputum samples for 19 respiratory tract infections (BioFire FilmArray Respiratory Panel 2.1) on the same day detected SARS-CoV-2 but no other viruses or bacteria causing respiratory tract infection (Table S2). The patient was readmitted to Osaka City General Hospital and treated with remdesivir from day 239. Remdesivir was administered at 200 mg/day on the first day and 100 mg/day from the second day onwards for 10 days in total, based on methods described in previous studies.^3^^,^^10^ On the second day of remdesivir administration (day 240), the fever subsided, and the chills, shivering, and malaise disappeared. Blood testing showed rapid decreases in the levels of CRP and IL-6 (Figure 1A, Table S3), and imaging tests showed the resolution of pneumonia (Figure 1B). The qRT-PCR testing of sputum samples for SARS-CoV-2 showed elevated Ct values, and the patient tested negative for the virus on day 243 (Figure 1A, Table S4). No SARS-CoV-2 was isolated from the sputum samples after the administration of remdesivir. The patient was discharged on day 248 after all of his symptoms had been resolved and the blood and imaging examination results had improved. No flare-ups have occurred for 9 months since his discharge, indicating that the treatment was successful. The patient was given intravenous immunoglobulin (IVIG) injections every month (Figure 1A, Table S5) and was not vaccinated against SARS-CoV-2 during the entire course of this study. Tests for the anti-SARS-CoV-2 spike (S) protein in the plasma and IVIG lots administered to the patient during the study period yielded negative results (Figure S1).

### Viral genome and proliferative capacity analysis

**Figure 2:**
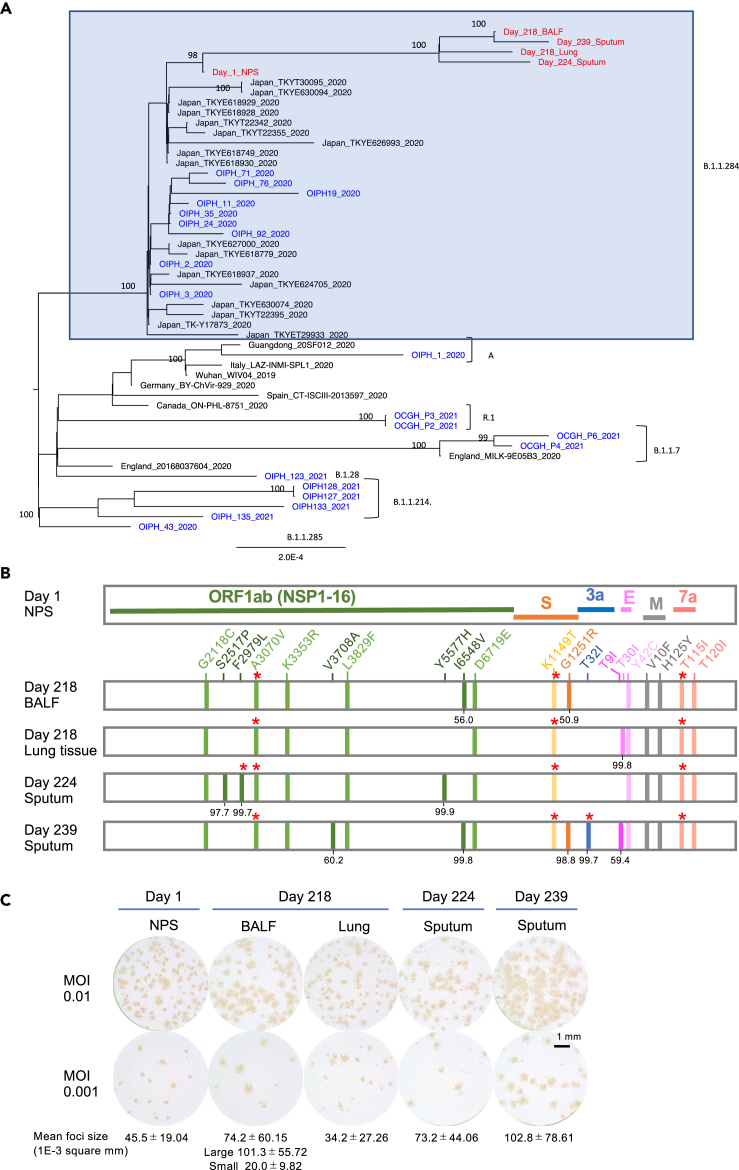
Whole-genome sequencing, phylogenetic relationships, and proliferative capacity of SARS-CoV-2 isolated from the patient (A) Phylogenetic tree of the viruses isolated from the patient on days 1, 218, 224, and 239 (red), along with representative global and local (Osaka, Japan) sequences. Viruses isolated from the patient and other local patients are shown in red and blue, respectively. (B) Locations of amino acid changes in the viruses isolated on days 218, 224, and 239 in the patient compared with those in the day-1 virus. The numbers below the mutations indicate the percentage of the mutation. ORF, open reading frame; NSP, non-structural protein; S, spike; *3a*, ORF3a; E, envelope; M, membrane; 7a, ORF7a. ∗ Amino acid changes involved in T-cell recognition. (C) Focus phenotypes of the isolated viruses (passage #1) were characterized in VeroE6/TMPRSS2 cells. MOI, multiplicity of infection. Scale bar depicted in (C) is 1 mm. See also Tables S4, S6, and S7.

Infectious viruses were isolated from cryopreserved specimens from a nasopharyngeal swab (NPS) on day 1, bronchoalveolar lavage fluid (BALF) and lung tissue on day 218, sputum on day 224, and fresh sputum on day 239 (Table S4). Whole-genome sequences were obtained from the five isolates and day-1 NPS specimen. A phylogenetic analysis showed that all of the viruses belonged to the clade B.1.1.284, which was a prevalent clade in Japan during the period in which the patient was infected. The isolated day-1 virus showed the same consensus sequence as the day-1 NPS specimen, and the other viruses formed a distinct cluster with the day-1 viruses (Figure 2A, Table S6). It should be noted that most of the viruses in Japan between days 218–239, during which the mutated viruses in the patient were isolated, belonged to clade B.1.1.7. In contrast, the viruses isolated from the patient belonged to B.1.1.284, which nearly disappeared between days 218–239 in Japan.^15^^,^^16^^,^^17^ These results demonstrate that the worsening of the condition of the patient after day 235 was due to a relapse of COVID-19, ruling out the possibility of reinfection with another virus. Nearly two-thirds of the mutations were non-synonymous (Table S7), suggesting the presence of positive selection pressure. Compared with the day-1 viruses, 11 amino acid changes and two synonymous nucleotide substitutions were commonly observed in all of the other isolates (Figure 2B). An additional two, one, three, and five amino acid changes were found in the isolates from the BALF and lung tissue from day 218 and those from days 224 and 239, respectively. Apart from the K3353R mutation in *ORF1ab*, these mutations have rarely been found in the CoV-GLUE database^18^ (Table S7), indicating a unique evolution of SARS-CoV-2 in this patient. These mutations were unevenly distributed in the viral genome, and the average numbers of mutations per 1,000 nucleotides in these viruses exceeded 1 in the regions of E (6.67), M (3.00), and ORF7a (6.89), whereas those in ORF1a (0.454), ORF1b (0.278), S (0.720), and ORF3a (0.303) were less than 1. There were no mutations in the ORF6, ORF8, and N regions. The evolution rate of SARS-CoV-2 in this patient was higher (9.37 × 10^−4^/site/year) than that reported in general populations (3.95–6.67 × 10^−4^/site/year) (Table 1).^19^^,^^20^^,^^21^^,^^22^^,^^23^ We calculated the SARS-CoV-2 evolution rate based on published sequence data from other immunocompromised patients^2^^,^^4^^,^^10^^,^^12^^,^^13^^,^^23^^,^^24^ and confirmed that SARS-CoV-2 evolves faster in immunocompromised hosts than in the general population (Table 1).

**Table 1:** Evolution rate of SARS-CoV-2 in the general population and immunocompromised hosts

General population		
Population	Reference	Evolution rate/site/year (95% CI)^a^
Africa	Motayo et al.^19^	4.13 × 10^−4^
USA	Wang et al.^20^	6.67 × 10^−4^
UK	Hill et al.^21^	4.6 × 10^−4^
GISAID till March 2020	Shen et al.^22^	3.95 × 10^−4^
GISAID	Chaguzaet al.^23^	5.83 × 10^−4^ (5.56–6.11 × 10^−4^)

Immunocompromised patient			
Deficiency	Reference	Disease	Evolution rate/site/year (95% CI)^a^
Primary	Present study	X-linked agammaglobulinemia	9.37 × 10^−4^ (9.10–9.65 × 10^−4^)^b^
	Avanzato et al.^2^	Chronic lymphocytic leukemia	12.1 × 10^−4^ (5.88–18.2 × 10^−4^)^b^
	Buckland et al.^10^	X-linked agammaglobulinemia	27.3 × 10^−4^ (22.5–32.0 × 10^−4^)^b^
Secondary	Choi et al.^4^	Severe antiphospholipid syndrome	17.0 × 10^−4^ (14.8–19.2 × 10^−4^)^b^
	Borges et al.^24^	Non-Hodgkin lymphoma	11.3 × 10^−4^^b^
	Kemp et al.^12^	Marginal B cell lymphoma	11.4 × 10^−4^ (8.19–14.6 × 10^−4^)^b^
	Williamson et al.^13^	Chronic lymphocytic leukemia	12.0 × 10^−4^ (8.61–15.4 × 10^−4^)^b^
	Chaguzaet al.^23^	Advanced lymphocytic leukemia and B-cell lymphoma	12.1 × 10^−4^ (10.7–13.4 × 10^−4^)

a 95% confidential intervals.

b Calculated in the present study.

The proliferative capacities of the isolated viruses were evaluated using a focus-forming assay. Passage #1 viruses isolated from the NPS specimen on day 1, BALF and lung tissue on day 218, and sputum on days 224 and 239 were inoculated in VeroE6/TMPRSS2 cells, which were seeded in 24-well plates, at infection multiplicities of 0.01 and 0.001. The focus sizes of the viruses were compared 1 day after infection (Figure 2C). The viruses isolated from the day-1 NPS specimen had small foci (mean size, 45.5 ± 19.04 × 10^−3^ mm^2^), whereas those isolated from the sputum on day 239 had large foci (mean size, 102.8 ± 78.61 × 10^−3^ mm^2^). Although the viruses isolated from the lung tissue on day 218 formed small foci (mean size, 34.2 ± 27.26 × 10^−3^ mm^2^), those isolated from the BALF on day 218 formed both large (mean size, 101.3 ± 55.72 × 10^−3^ mm^2^) and small foci (mean size, 20.0 ± 9.82 × 10^−3^ mm^2^), suggesting that these isolates comprised a mixture of more than one viral clone. The viruses isolated from the sputum on day 224 formed medium-sized foci (mean size, 73.2 ± 44.06 × 10^−3^ mm^2^). Although several factors, including the binding and destruction of the receptor of the viruses, can affect focus or plaque sizes,^25^^,^^26^ it is generally believed that the sizes of foci are correlated with the proliferative capacities of viruses. These results suggest that SARS-CoV-2 evolved with an increased proliferative capacity in this patient.

### The T32I mutation in ORF3a evades T-cell recognition

**Figure 3:**
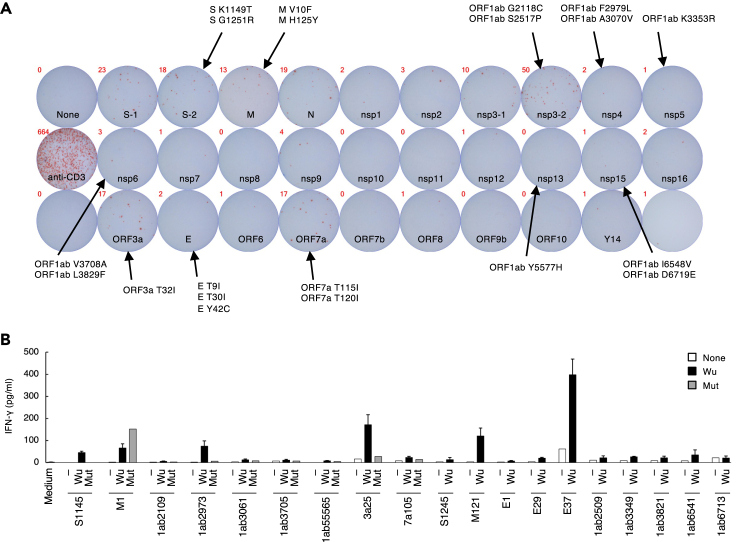
SARS-CoV-2 epitope recognition by peripheral blood mononuclear cells (PBMCs) from the patient (A) Reaction of PBMCs to the peptide pools derived from proteins of the SARS-CoV-2 Wuhan strain. PBMCs collected on day 308 were stimulated with peptide pools for 48 h, and IFN-γ expression was determined using an ELISPOT assay. SARS-CoV-2 proteins with mutations detected in the patient are indicated. (B) Concentrations of IFN-γ in the supernatant of PBMCs stimulated with SARS-CoV-2 peptides. PBMCs collected from the patient on day 324 were stimulated with the indicated individual peptides from the SARS-CoV-2 Wuhan-Hu-1 strain (Wu) for 12 days before being washed and re-stimulated with nothing (−), the same Wu peptides, or corresponding mutant peptides (Mut) for 20 h. Error bars indicate mean ± SD of duplicates. Data are representative of three independent experiments. See also Figure S2 and Table S8.

To profile the immune response against SARS-CoV-2 in the patient, the T-cell response against SARS-CoV-2 antigens was first examined. Peripheral blood mononuclear cells (PBMCs) were stimulated with peptide pools derived from proteins of the SARS-CoV-2 Wuhan-Hu-1 strain (Wu) in an ELISPOT assay (Figure 3A). Interferon (IFN)-γ-expressing cells were mainly observed when the PBMCs were stimulated with structural spike (S), membrane (M), nucleocapsid (N) proteins, accessory factors (ORF3a and ORF7a), and a non-structural protein (NSP3), suggesting that T-cell epitopes were mostly located in these proteins. These epitope hotspots included almost half of the mutations detected in the patient, implying that they were selected under pressure from the host T-cell response.

To further investigate the pivotal mutations that aggravated the infection, the T-cell response to single epitopes covering the mutated regions was examined. PBMCs from the patient were expanded with peptides from Wu that covered the mutated regions (Figure 3A) before they were re-stimulated with the same or corresponding mutant (mut) peptides (Table S8). The S1145, ORF1ab2973, and ORF3a25 Wu peptides induced greater IFN-γ expression than their corresponding mutant peptides (Figure 3B). In addition, a decreased expression of IFN-γ induced by ORF1ab3061 mut and ORF7a105 T115I mut peptides was repeatedly observed in three experiments, indicating that mutations in these five regions were likely involved in the immune escape of the viral variants.

**Figure 4:**
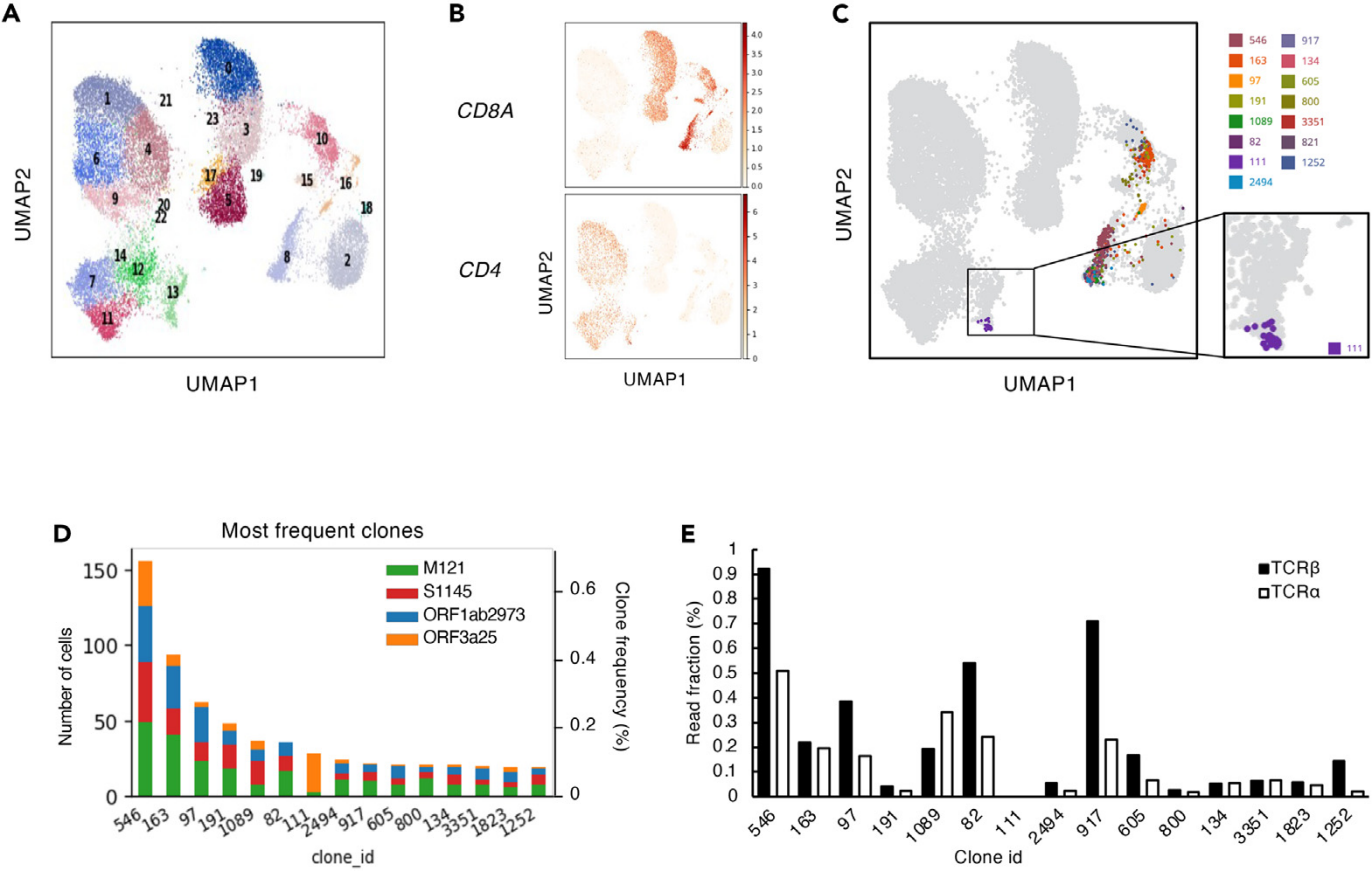
CD4 T-cell clone-111 was specifically expanded following ORF3a25 stimulation (A) Results of single-cell TCR- and RNA-seq analyses. PBMCs collected from the patient on day 324 were stimulated with S1145, ORF1ab2973, ORF3a25, or M121 Wu peptides in individual wells for 15 days before the CD3^+^T cells were sorted, hashtagged, mixed, and analyzed by single-cell TCR- and RNA-seq. Each dot in the UMAP plot corresponds to a single cell and is colored according to the Leiden cluster. (B) mRNA expression of *CD4* and *CD8A* in the single cells from the single-cell analyses described in (A). (C) The top-15 most frequent T-cell clones detected in the single-cell analyses described in (A) are shown in the UMAP plot. (D) Clone size (left yaxis), clone fraction (right yaxis), and origin of the top-15 frequent clones detected in the single-cell analyses shown in (A). (E) Frequencies of the 15 clones shown in (D) before stimulation were determined by bulk TCR-seq using unstimulated PBMCs. The read fractions of the TCR-α and-β sequences are shown. See also Tables S8 and S9.

**Figure 5:**
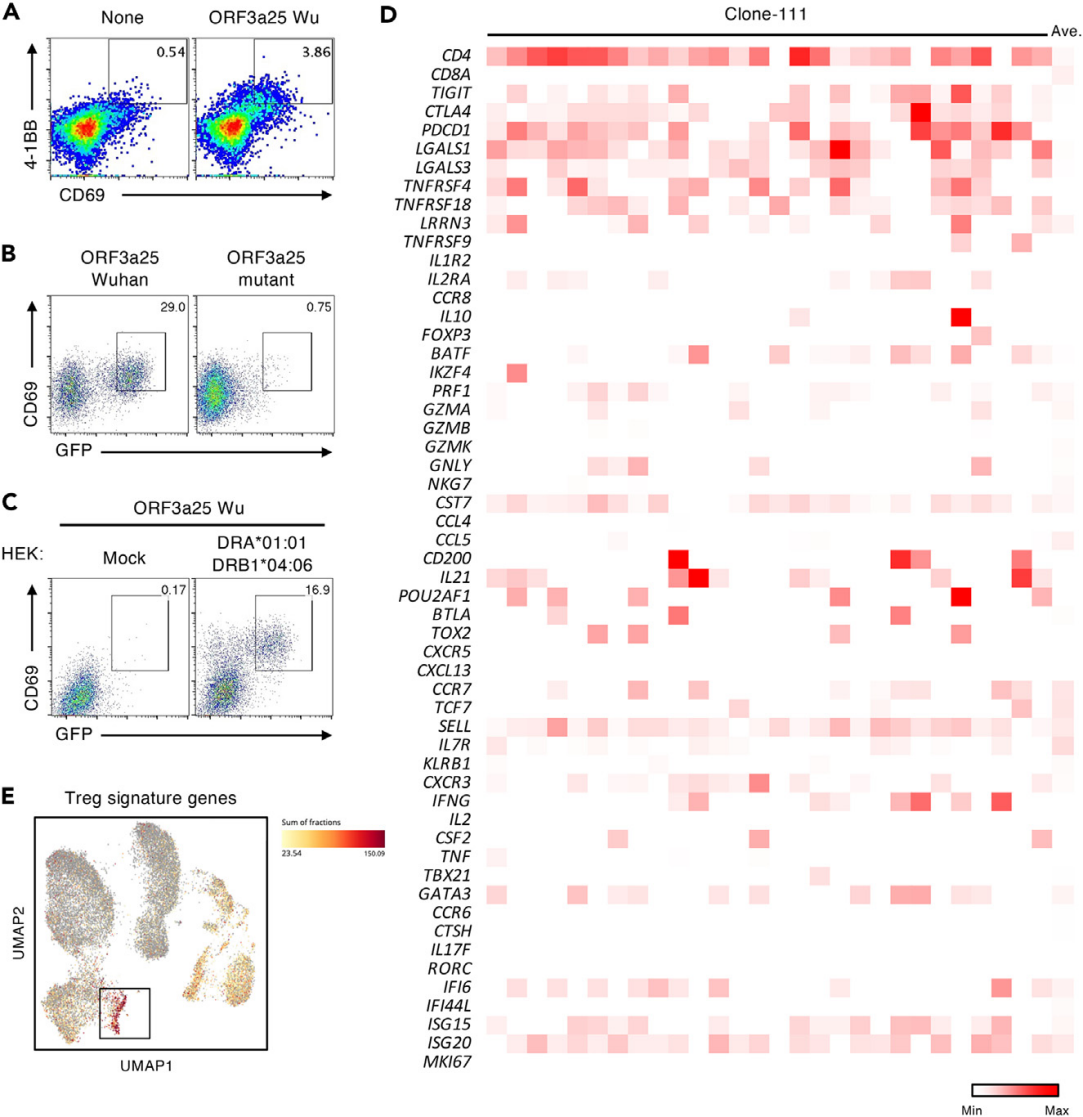
The T32I mutation in ORF3a evades recognition by a CD4^+^ T-cell clone (A) Percentages of CD69+4-1BB+ populations in CD4^+^ T-cell gates of PBMCs stimulated with the ORF3a25 Wu peptide for 12 days before being washed and re-stimulated with nothing (None) or the same peptide (ORF3a25 Wu) for 24 h. (B) Reporter cells expressing the TCRs of clone-111 were stimulated with ORF3a25 Wu or Mut peptides in the presence of autologous PBMCs and analyzed for GFP and CD69 expression. (C) The restricting human leukocyte antigen of clone-111 was DRB1∗04:06. Reporter cells expressing the TCRs of clone-111 were stimulated with the ORF3a25 Wu peptide in the presence of HEK293T cells transfected with nothing or DRA∗01:01-DRB1∗04:06-expressing plasmids and were analyzed for GFP and CD69 expression. (D) mRNA expression of the characteristic genes of CD4^+^ T-cell subsets in clone-111 cells. (E) Expression of Treg signature genes (*FOXP3*, *CTLA4*, *DUSP4*, *IL2RB*, *STAM*, *DOK2*, and *TNFRSF1B*) in the single cells in Figure 4A. The black box indicates the cluster containing clone-111. The sum of fractions of the Treg signature genes was calculated using BBrowser and is shown as a heatmap. Data are representative of three independent experiments (A–C). See also Tables S1 and S8.

To further elucidate the characteristics of the T cells targeting these critical regions, T cells were sorted after being stimulated with S1145, ORF1ab2973, ORF3a25, or M121 Wu peptides, pooled, and analyzed using single-cell T-cell receptor (TCR)- and RNA-sequencing analyses (Figure 4A). The TCR sequencing results showed that some of the clonotypes had higher frequencies after the *in vitro* stimulations within both the CD4^+^ and CD8^+^ clusters (Figure 4B). Within the top-15 frequent T-cell clones identified in the single-cell analyses, 14 clones were located in clusters reflecting CD8^+^ CTL-like cells (Figure 4C). The remaining clone-111 comprised CD4^+^ cells (Figure 4C) and was predominantly from ORF3a25-stimulated cells (Figure 4D), suggesting that this clone was preferentially expanded by the stimulation of ORF3a25. All of the top 15 frequent clones, except clone-111, were detected in the PBMCs of the patient before the *in vitro* stimulation (Figure 4E), suggesting that clone-111 had a low frequency before the stimulation and became dominant afterward. Consistent with this, the ORF3a25 Wu peptide was found to activate the *in vitro*-expanded CD4^+^ T cells upon re-stimulation (Figure 5A). To confirm the epitope of clone-111, TCR-α and-β of clone-111 were reconstituted in a TCR-deficient reporter cell line. The reporter cells were activated by the ORF3a25 Wu peptide in the presence of autologous antigen-presenting cells, but the T32I mutant peptide lost antigenicity (Figure 5B). Of the human leukocyte antigen class II genes in the patient, DRB1∗04:06 was identified as a restricting allele for ORF3a epitope recognition (Figure 5C). These results indicate that the T32I mutation in ORF3a resulted in the evasion of T-cell recognition.

Clone-111, which was detected as 28 different barcoded cells in a CD4^+^ cluster (Figure 4C), showed the gene signatures of highly activated cells (Figure 5D). These cells also expressed genes related to heterogeneous functions, such as IFN-γ, IL-21, PRF1, and IL-10 (Figure 5D). Notably, clone-111 was located in the cluster with a high expression of T regulatory (Treg) signature genes (Figure 5E),^27^ although most of the clone-111 cells did not highly express *FOXP3*. These observations suggest the helper function, cytotoxicity, and regulatory activity of this clonotype during infection.^20^^,^^27^^,^^28^^,^^29^ The finding that SARS-CoV-2 escaped the CD4^+^ clonotype with multiple potentials in the absence of humoral immunity further suggests a previously unclear role of CD4^+^ cells in the immune protection against COVID-19.

## Discussion

We evaluated a patient with XLA in whom SARS-CoV-2 evolved uniquely for 239 days. During the whole infection period, 20 non-synonymous mutations were acquired by the virus, and many of these mutations were previously unreported (GISAID, https://www.gisaid.org). Among them, the ORF3a T32I mutation evaded recognition by CD4^+^ T cells with multi-functional characteristics, such as cytotoxic and regulatory function. Indeed, infection was controlled for 8 months when the original ORF3a epitope (ORF3a T32) was preserved, suggesting that T cells recognizing this epitope have a potent protective function even in the absence of humoral immunity. Consistently, when the mutation was acquired on day 239, the patient suffered severe deterioration with drastically increased levels of IL-6 and CRP and the sputum viral load. Thus, this variant is considered to acquire escape capacity from protective T cells, which results in viral expansion and the subsequent exacerbation of pneumonia in the patient.

The patient was given immunoglobulin every month because no normal mature B cells were present in his peripheral blood. The globulin products administered during the observation period were manufactured from blood collected before the emergence of COVID-19 (Table S5). Therefore, no SARS-CoV-2-specific antibodies were present in the serum of the patient (Figure S1). This case illustrates the pathophysiology of SARS-CoV-2 infection in the absence of specific humoral immunity.

Several precedents for SARS-CoV-2 evolution in immunocompromised patients with partially attenuated humoral responses have been described.^4^^,^^12^^,^^24^^,^^30^^,^^31^^,^^32^^,^^33^ SARS-CoV-2 evolved faster in these patients (Table 1), and its mutations showed a certain degree of similarity to those in several variants of concern, such as B.1.17, B.1.135, and P1, because these viruses carry deletions in the N-terminal domain and E484K or N501Y substitutions in the spike protein. The observed SARS-CoV-2 mutations in our patient, however, have rarely been reported, except for the K3353R mutation in *ORF1ab*, possibly due to a near-complete lack of humoral immune responses.

In the absence of humoral immunity, cellular immunity should exert protective effects against pathogens. The 28 CD4^+^ T cells from clone-111 had the same TCRs but showed the characteristics of different cell types, such as T follicular helper (Tfh), CD4 CTL, and Treg cells (Figures 5D and 5E).^20^^,^^27^^,^^28^^,^^29^ These results suggest that this CD4^+^ T-cell clonotype may exhibit functional plasticity and balance the T-cell responses in the protective immunity against SARS-CoV-2 infection. It has been reported that epitope affinity may affect the fate of T-cell clonotypes.^34^^,^^35^ However, in the case evaluated in our study, the development of heterogeneous T-cell types did not seem to be restricted by the epitope.

In our patient, CD4-mediated responses were likely to be dominant, because, in addition to ORF3a, four more mutations (ORF1ab F2979L in NSP4, ORF1ab L3829F in NSP6, K1149T in S, and T1155I in ORF7a) escaped from CD4^+^ T-cell recognition, whereas no mutations lost antigenicity to CD8^+^ T cells (Figure S2). Several mutant peptides, such as M1, ORF1ab2509, and ORF1ab3061, activated more CD4^+^ T cells than the corresponding Wu peptides did. These mutations may have occurred due to viral fitness, which warrants further investigation. The reason for the dominance of CD4-mediated responses is unclear at present. Nevertheless, it is known that MHC class II deficiency, but not MHC class I deficiency, incapacitates the immune system during viral infection,^36^which suggests an indispensable role of CD4^+^ T cells in antiviral immunity. Therefore, the identification of *de novo* mutations that avoid T-cell recognition in the absence of protective antibodies may help to predict future “T-escaping” variants when broad neutralization antibodies prevail in developing vaccines.^37^^,^^38^

The ORF3a protein is unique for the coronavirus subgenus *Sarbecovirus*, which includes SARS-CoV and SARS-CoV-2. ORF3a works as a viroporin, assisting the entry and release of the virus.^39^ Because ORF3a has multiple roles in the life cycle of SARS-CoV-2 and the induction of host immune responses, it is hard to predict the influence of the T32I mutation in ORF3a. Nevertheless, given that we observed a deteriorated infection after the occurrence of this mutation, we suspect that this mutation does not disrupt or even benefit ORF3a function. The replicative speed of this mutant virus isolated on day 239 was higher than that of the day-1 virus without the ORF3a mutation.

As shown in the results in Figure 2C, the day-1 virus formed small foci, whereas the day-239 virus formed large foci. Like the day-1 virus, the day-218 lung tissue virus formed small foci, but the day-218-BALF virus formed both small and large foci. The day-239 and day-218 BALF viruses shared ORF1ab I6548V and S G1251R mutations, both of which were absent in the other viruses. These mutations were detected in approximately half of the day-218-BALF virus population and nearly all of the day-239 virus population (Figure 2B). These results suggest that ORF1ab I6548V and/or S G1251R mutations may have been involved in the increased proliferative ability of the virus. This is because the S protein plays an important role in viral entry into cells,^40^ and ORF1ab 6548 is located at NSP15, which is an endoribonuclease important for viral replication.^41^

The administration of remdesivir dramatically improved the symptoms, inflammatory markers, and imaging test results of the patient, and the shedding of infectious viral particles stopped immediately. Remdesivir induced strong antiviral activity in the patient, which was consistent with results from a previous report. SARS-CoV-2 may therefore be eliminated by remdesivir alone without SARS-CoV-2-specific antibodies.^42^

In conclusion, SARS-CoV-2 evolved to evade recognition by CD4^+^ T cells and increased in its proliferative capacity during the persistent infection in our reported patient, which raises the possibility of the emergence of such variants in future even in immunocompetent patients, particularly when the escape from neutralizing antibodies is not sufficient for immune evasion. Thus, the control of chronic SARS-CoV-2 infection would also be beneficial for the prevention of virulent variants that evade such multi-functional T cells. In particular, immunocompromised patients should be treated with available antiviral drugs to prevent the emergence of public health-threatening mutants. We recently reported that non-spike epitopes recognized by CD8^+^ T cells contribute to the protection.^43^ Given the importance of multi-functional CD4^+^ T cells for protection in this study, the epitopes of such CD4^+^ T cells can be promising candidate antigens for the next-generation vaccines, because these epitopes are conserved in currently-reported VOCs. Extensive investigation of variants and T cell responses is therefore a crucial measure for the sustainable control of SARS-CoV-2 worldwide.

### Limitations of the study

Despite the importance of our findings, there were several limitations in this study. These included the number of cases, because we could not investigate other patients with XLA who were also infected with SARS-CoV-2 as such cases are rare. Nevertheless, we were able to show the accelerated evolution of SARS-CoV-2 in the case evaluated herein as well as in other reported immunocompromised cases. In addition, the clinical specimens, especially the PBMCs that were used for the T-cell analysis, were mainly collected after day 210, and we failed to obtain evidence for the immune evasion of some of the mutations that were identified. We were, however, able to identify a*de novo*mutation that evaded the recognition of dominant T cells in the patient with XLA.

## STAR★Methods

### Key resources table

**Table:** 

REAGENT or RESOURCE	SOURCE	IDENTIFIER
**Antibodies**		

Mouse monoclonal anti Coronavirus (SARS-CoV-2 and SARS-CoV NP)	EastCoast Bio	Cat#HM1054
Rabbit polyclonal anti-mouse IgG (whole molecule)	MP Biomedicals	Cat#55436
Goat polyclonal anti-rabbit IgG (whole molecule)	MP Biomedicals	Cat#55602
Rabbit peroxidase-anti-peroxidase	Jackson Immuno Research	Cat#323-005-024; RRID: AB_2315781
Peroxidase-conjugated AffiniPure Alpaca Anti-Human IgG (H+L)	Jackson Immuno Research	Cat#609-035-213; RRID: AB_2721849
Anti-human CD3e FITC	BioLegend	Cat#300306; RRID: AB_314042
Anti-human CD8a PE	BioLegend	Cat#301008; RRID: AB_314126
Anti-human CD4 AF-700	BioLegend	Cat#344621; RRID: AB_2563149
Anti-human CD69 PE	BioLegend	Cat#310906; RRID: AB_314841
Anti-human CD69 PECy7	BioLegend	Cat#310911; RRID: AB_314846
Anti-human CD137 APC	BioLegend	Cat#309810; RRID: AB_830672
Anti-human Hashtags 1	BioLegend	Cat#394661; RRID: AB_2801031
Anti-human Hashtags 2	BioLegend	Cat#394663; RRID: AB_2801032
Anti-human Hashtags 3	BioLegend	Cat#394665; RRID: AB_2801033
Anti-human Hashtags 4	BioLegend	Cat#394667; RRID: AB_2801034
Anti-mouse CD69 APC	BioLegend	Cat#104514; RRID: AB_492843

**Bacterial and viral strains**		

hCoV–19/Japan/RIMD–DVI–C10B/2021	This paper	GISAD: EPI_ISL_4935777
hCoV–19/Japan/RIMD–DVI–C10L/2021	This paper	GISAD: EPI_ISL_4935949
hCoV–19/Japan/RIMD–DVI–C16/2021	This paper	GISAD: EPI_ISL_4936095
hCoV–19/Japan/RIMD–DVI–H06/2020	This paper	GISAD: EPI_ISL_4936243
hCoV–19/Japan/RIMD–DVI–C31/2021	This paper	GISAD: EPI_ISL_4936533

**Biological samples**		

Patient nasopharyngeal swabs	This paper	Osaka City General Hospital
Patient bronchoalveolar lavage fluid	This paper	Osaka City General Hospital
Patient sputum	This paper	Osaka City General Hospital
Patient saliva	This paper	Osaka City General Hospital
Patient lung tissue obtained by biopsy	This paper	Osaka City General Hospital
Patient suction phlegm	This paper	Osaka City General Hospital
Patient blood	This paper	Osaka City General Hospital
Patient urine	This paper	Osaka City General Hospital
Human intravenous immunoglobulin (Pirivigen 10% I.V. Drip Infusion 10 g/100 mL)	CSL Behring	23100AMX00288
Healthy donor sera	This paper	Osaka City General Hospital
First WHO International Standard for anti-SARS-CoV-2 immunoglobulin (human)	NIBSC	Code: 20/136

**Chemicals, peptides, and recombinant proteins**		

3,3′-Diaminobenzidine tetrahydrochloride	Sigma-Aldrich	Cat#D5905
Hydrogen peroxide	FUJIFILM Wako Pure Chemical Corporation	Cat#084-07441
SARS-CoV-2 Spike Protein (S1+S2 ECD, His tag)	Sino Biological	Cat#40589-V08B1
TMB Substrate Kit	Thermo Scientific	Cat#34021
Non-Essential Amino Acid Solution	Gibco-BRL	Cat#11140-050
Sodium pyruvate	Gibco-BRL	Cat#06977-34
Synthesized peptides	Genscript	Customized
PepMix™ SARS-CoV-2 (Spike Glycoprotein)	JPT	PM-WCPV-S-2
PepMix™ SARS-CoV-2 (VME1)	JPT	PM-WCPV-VME
PepMix™ SARS-CoV-2 (NCAP)	JPT	PM-WCPV-NCAP
PepMix™ SARS-CoV-2 (Nsp1)	JPT	PM-WCPV-NSP01-1
PepMix™ SARS-CoV-2 (Nsp2)	JPT	PM-WCPV-NSP02-1
PepMix™ SARS-CoV-2 (Nsp3)	JPT	PM-WCPV-NSP03-1
PepMix™ SARS-CoV-2 (Nsp4)	JPT	PM-WCPV-NSP04-1
PepMix™ SARS-CoV-2 (Nsp5)	JPT	PM-WCPV-NSP5-1
PepMix™ SARS-CoV-2 (Nsp6)	JPT	PM-WCPV-NSP06-1
PepMix™ SARS-CoV-2 (Nsp7)	JPT	PM-WCPV-NSP07-1
PepMix™ SARS-CoV-2 (Nsp8)	JPT	PM-WCPV-NSP08-1
PepMix™ SARS-CoV-2 (Nsp9)	JPT	PM-WCPV-NSP09-1
PepMix™ SARS-CoV-2 (Nsp10)	JPT	PM-WCPV-NSP10-1
PepMix™ SARS-CoV-2 (C+Nsp11)	JPT	PM-WCPV-CLEAVE+Nsp11
PepMix™ SARS-CoV-2 (Nsp12)	JPT	PM-WCPV-NSP12-1
PepMix™ SARS-CoV-2 (Nsp13)	JPT	PM-WCPV-NSP13-1
PepMix™ SARS-CoV-2 (Nsp15)	JPT	PM-WCPV-NSP15-1
PepMix™ SARS-CoV-2 (Nsp16)	JPT	PM-WCPV-NSP16-1
PepMix™ SARS-CoV-2 (AP3A)	JPT	PM-WCPV-AP3A
PepMix™ SARS-CoV-2 (VEMP)	JPT	PM-WCPV-VEMP
PepMix™ SARS-CoV-2 (NS6)	JPT	PM-WCPV-NS6
PepMix™ SARS-CoV-2 (NS7a)	JPT	PM-WCPV-NS7A
PepMix™ SARS-CoV-2 (NS7B)	JPT	PM-WCPV-NS7B
PepMix™ SARS-CoV-2 (NS8)	JPT	PM-WCPV-NS8
PepMix™ SARS-CoV-2 (ORF9B)	JPT	PM-WCPV-ORF9B
PepMix™ SARS-CoV-2 (ORF10)	JPT	PM-WCPV-ORF10
PepMix™ SARS-CoV-2 (Y14)	JPT	PM-WCPV-Y14
Recombinant human IL-2	Peprotech	Cat#212-12
Recombinant human IL-15	Peprotech	Cat#200-15
Recombinant human granulocyte–macrophage colony–stimulating factor (GM-CSF)	Peprotech	Cat#300-03
Recombinant human IL-4	Peprotech	Cat#200-04
Recombinant human IL-7	BioLegend	Cat#581902

**Critical commercial assays**		

QIAamp Viral RNA Mini kit	QIAGEN	Cat#52906
One Step PrimeScript™ RT-PCR Kit	Takara	Cat# RR064A
GenNext RamDA-seq Single Cell Kit	TOYOBO	Cat#RMD-101
Nextera XT DNA Library Prep Kit	Illumina	Cat#FC-131-1096
Nextera XT Index Kit v2	Illumina	Cat#FC-131-2001
LEGENDplex Human CD8/NK Panel (13-plex)	BioLegend	Cat#740267
Human interferon-γ/IL-4 Double-Color ELISPOT	ImmunoSpot	Cat#hIFNgIL4-1M/10
Human CD14 MicroBeads	Miltenyi Biotec	Cat#130-050-201
QIAzol	Qiagen	Cat#79306

**Deposited data**		

Single-cell TCR- and RNA-sequencing data	This paper	GEO datasets: GSE190895
hCoV-19/Japan/RIMD-DVI-C26H/2021 (SARS-CoV-2 sequence)	This paper	GSAID: EPI_ISL_4929525
hCoV-19/Japan/RIMD-DVI-C26W/2021 (SARS-CoV-2 sequence)	This paper	GSAID: EPI_ISL_4930839
hCoV-19/Japan/RIMD-DVI-D09/2021 (SARS-CoV-2 sequence)	This paper	GSAID: EPI_ISL_4931056
hCoV-19/Japan/RIMD-DVI-D21/2021 (SARS-CoV-2 sequence)	This paper	GSAID: EPI_ISL_4931846
hCoV-19/Japan/OIPH14/2020 (SARS-CoV-2 sequence)	This paper	GSAID: EPI_ISL_4932136
hCoV-19/Japan/OIPH16/2020 (SARS-CoV-2 sequence)	This paper	GSAID: EPI_ISL_4932550
hCoV-19/Japan/OIPH21/2020 (SARS-CoV-2 sequence)	This paper	GSAID: EPI_ISL_4932712
hCoV-19Japan/OIPH29/2020 (SARS-CoV-2 sequence)	This paper	GSAID: EPI_ISL_4932860
hCoV-19/Japan/OIPH34/2020 (SARS-CoV-2 sequence)	This paper	GSAID: EPI_ISL_4933015
hCoV-19/Japan/OIPH6/2020 (SARS-CoV-2 sequence)	This paper	GSAID: EPI_ISL_4933271
hCoV-19/Japan/OIPH51/2020 (SARS-CoV-2 sequence)	This paper	GSAID: EPI_ISL_4933542
hCoV-19/Japan/OIPH54/2020 (SARS-CoV-2 sequence)	This paper	GSAID: EPI_ISL_4933741
hCoV-19Japan/OIPH61/2020 (SARS-CoV-2 sequence)	This paper	GSAID: EPI_ISL_4933859
hCoV-19/Japan/OIPH1/2021 (SARS-CoV-2 sequence)	This paper	GSAID: EPI_ISL_4933990
hCoV-19/Japan/OIPH25/2020 (SARS-CoV-2 sequence)	This paper	GSAID: EPI_ISL_4934247
hCoV-19/Japan/OIPH28/2020 (SARS-CoV-2 sequence)	This paper	GSAID: EPI_ISL_4934751
hCoV-19/Japan/OIPH5/2021 (SARS-CoV-2 sequence)	This paper	GSAID: EPI_ISL_4934873
hCoV-19/Japan/OIPH6/2021 (SARS-CoV-2 sequence)	This paper	GSAID: EPI_ISL_4934996
hCoV-19/Japan/OIPH10/2021 (SARS-CoV-2 sequence)	This paper	GSAID: EPI_ISL_4935185
hCoV-19/Japan/OIPH12/2021 (SARS-CoV-2 sequence)	This paper	GSAID: EPI_ISL_4935429

**Experimental models: Cell lines**		

VeroE6/TMPRSS2	JCRB cell bank	Cat#JCRB1819
NFAT-GFP reporter mouse T cell hybridoma	Osaka University	Matsumoto et al.^44^

**Oligonucleotides**		

NIID_2019-nCOV_N_F2 AAATTTTGGGGACCAGGAAC	This paper	https://www.niid.go.jp/niid/images/epi/corona/2019-nCoVmanual20200217-en.pdf
NIID_2019-nCOV_N_R2 TGGCAGCTGTGTAGGTCAAC∗	This paper	https://www.niid.go.jp/niid/images/epi/corona/2019-nCoVmanual20200217-en.pdf
NIID_2019-nCOV_N_P2 FAM-ATGTCGCGCATTGGCATGGA-BHQ	This paper	https://www.niid.go.jp/niid/images/epi/corona/2019-nCoVmanual20200217-en.pdf

**Recombinant DNA**		

TCR sequences of clone-111	IDT	Customized

**Software and algorithms**		

Cutadapt	Martin^45^	https://cutadapt.readthedocs.io/en/stable/index.html
bwa	Li and Durbin^46^	http://bio-bwa.sourceforge.net/
Mutect2 (GATK 4.1.2.0)	Cibulskis et al.^47^	https://gatk.broadinstitute.org/hc/en-us/articles/360037593851-Mutect2
snpEff	Cingolani et al.^48^	http://pcingola.github.io/SnpEff/
Haplotype Caller (GATK 4.1.2.0)	Poplin et al.^49^	https://gatk.broadinstitute.org/hc/en-us/articles/360037225632-HaplotypeCaller
bcftools ver1.9	Li^50^	https://samtools.github.io/bcftools/bcftools.html
W-IQ-TREE	Trifinopoulos et al.^51^	http://iqtree.cibiv.univie.ac.at
ModelFinder	Kalyaanamoorthy et al.^52^	http://iqtree.org/
Ultrafast bootstrap	Hoang et al.^53^	http://iqtree.org/

**Other**		

2019-nCoV one-step RT-qPCR Positive control RNA for N2 set	Nihon Gene Research Laboratories, Inc.	Cat#JP-NN2-PC
Human AB serum (off the clot)	Gemini Bio	Cat#100-318

### Resource availability

#### Lead contact

Requests for further information, resources, and reagents should be directed to and will be fulfilled by the Lead Contact, Tatsuo Shioda (shioda@biken.osaka-u.ac.jp).

#### Materials availability

This study did not generate new reagents.

### Experimental model and subject details

#### Ethical considerations

Written informed consent for the present study was obtained from the patient. The protocols of the present study were approved by the Local Incorporated Administrative Agency Osaka City Hospital Organization and Osaka City General Hospital Research Ethics Review Board (approval numbers: 2105017 and 2107048) and the institutional review board of the Research Institute for Microbial Diseases, Osaka University (approval number: 2021–4). The authors vouch for the accuracy and completeness of the data in this report.

#### COVID-19 patient

The patient, a 25-year-old man, experienced repeated bacterial infections from approximately 8 months of age and was genetically diagnosed with X-linked agammaglobulinemia (XLA) due to an exon 6 skip mutation at 11 months. He has since been given immunoglobulin every month (Table S5) but no prophylactic antibiotics. He developed fever at the end of July 2020 and was diagnosed with COVID-19 based on a positive result from a quantitative real-time reverse transcription polymerase chain reaction (qRT-PCR) test for SARS-CoV-2 in early August.

#### Cell lines

VeroE6/TMPRSS2 cells were obtained from the JCRB cell bank (cat. JCRB1819). The cells were maintained at 37 °C and with 5% CO_2_ in Dulbecco’s Modified Eagle’s Medium (DMEM) supplemented with 10% fetal bovine serum, 1 mM L-glutamine, 100 units/mL penicillin, and 100 μg/mL streptomycin. Mouse T-cell hybridoma with a nuclear factor of activated T cells (NFAT)-green fluorescent protein (GFP) reporter gene^44^ were maintained at 37 °C and with 5% CO_2_ in RPMI 1640 medium supplemented with 10% fetal bovine serum, penicillin, streptomycin, and 2-mercaptoethanol.

### Method details

#### Sample preparation for PCR testing and viral infectivity studies

Fresh samples (obtained within 24 h of sampling) and those that had been cryopreserved at 80 °C were obtained from a nasopharyngeal swab (NPS), bronchoalveolar lavage fluid, sputum and saliva, lung tissue obtained by a biopsy, and suction phlegm and used for PCR tests and viral infectivity studies. Before extracting the RNA, the sputum and saliva samples were diluted two- or four-fold using phosphate-buffered saline (PBS). A lung tissue sample was homogenized by adding a four-fold volume of PBS.

#### PCR test

A qRT-PCR was performed as previously described,^54^ with minor modifications. In brief, the samples were centrifuged at 10,000 rpm for 30 s to remove insoluble material. Viral RNA was extracted from 140 μL of the supernatant using the QIAamp Viral RNA Mini Kit (QIAGEN) according to the manufacturer’s instructions. Assays for the qRT-PCR were performed using the OneStep PrimeScript™ RT-PCR Kit (Takara Bio) with the N2 set of primers and a probe designed according to the methods published by the National Institute of Infectious Diseases, Japan. The conditions for the qRT-PCR were as follows: 52 °C for 5 min and 95 °C for 10 s, followed by 40 cycles at 95 °C for 5 s and 60 °C for 30 s using the QuantStudio™ 3 Real-Time PCR System (Life Technologies).

#### In-house ELISA of the anti-SARS-CoV-2 spike (S) protein antibody

A 96-well flat-bottom microplate was coated with 0.1 μg/well of the S protein (Sino Biological) in 50 μL/well of carbonate-bicarbonate buffer (Sigma-Aldrich) and incubated at 4 °C overnight. The plate was washed with 0.05% Tween 20 in PBS (PBS-T) and blocked with 300 μL/well of 25% BlockAce for 2hat 37 °C. After washing the plate with PBS-T, 50 μL/well of diluted patient plasma (1:100), control sera, and diluted 10% intravenous immunoglobulin (1:1,000) were added and incubated for 1hat 37 °C. After washing the sample three times with PBS-T, 50 μL/well of diluted peroxidase-conjugated alpaca anti-human IgG (H+L; 1:50,000) (Jackson ImmunoResearch) was added and incubated for 1hat 37 °C. After washing the sample three times, 50 μL/well of 3,3',5,5'-tetramethylbenzidine solution (TMB substrate kit; Thermo Scientific) was added and incubated for 5minat 25 ± 2 °C. The peroxidase reaction was stopped with 50 μL/well of 1 M sulfuric acid. The optical density at 450 nm was measured using a Multigrading Microplate Reader (SH-9500Lab; Corona). Antibody titers were calculated in units of binding antibody unit (BAU)/mL with calibrators assigned to the first WHO international standard for anti-SARS-CoV-2 immunoglobulin from the National Institute for Biological Standards and Control (code 20/136) as 1,000 BAU/mL.^55^^,^^56^

#### Focus-forming assay

The level of viral infectivity in the clinical specimens was measured using a focus-forming assay as previously described.^54^ In brief, VeroE6/TMPRSS2 cells were seeded to 24-well plates (2 x10^5^ cells/well) 1 day before the experiment. The medium was removed, and the cell monolayers in each well were inoculated with 100 μL of samples that had been serially diluted 10-fold. After 30 min, the samples were removed and washed three times with serum-free Minimum Essential Medium (MEM). The cells were covered with 0.5 mL of DMEM containing 1% carboxymethyl cellulose, 2% fetal bovine serum, and antibiotics (200 units/mL penicillin, 200 μg/mL streptomycin, and 0.25 μg/mL amphotericin B). Eighteen hours after being inoculated, the cells were fixed with 10% formalin for 30minat room temperature. They were then washed three times with PBS and fixed in absolute ethanol for 5minat room temperature. To visualize the virus-infected cells, the peroxidase-anti-peroxidase (PAP) technique was applied. Each well was treated for 40minat room temperature with 400 μL of each antibody in the following order: anti-SARS/SARS-2 NP mouse monoclonal antibody (EastCoast Bio) diluted to 1 μg/mL; anti-mouse rabbit immunoglobulin (Ig) G antibody (MP Biomedicals, Irvine, CA) diluted to 1:1,000; anti-rabbit goat IgG antibody (MP Biomedicals) diluted to 1:500; and rabbit PAP (Jackson Immuno Research) diluted to 1:200. After each reaction, the cells were washed three times with PBS. In the final step, the peroxidase reaction was developed for approximately 5 min using 0.01% H_2_O_2_ and 0.3 mg/mL of 3-3'-diaminobenzidine tetrahydrochloride in PBS. The cells were then washed with tap water and dried, and the stained foci were macroscopically counted. Viral infectivity was expressed as focus-forming units (FFUs). Focus sizes were measured using an ELISPOT analyzer (ImmunoSpot S6 VERSA Analyzer; M&S TechnoSystems, Inc., Japan).

#### Viral isolation

Viral isolation from the clinical specimens was performed as previously described.^54^ In brief, VeroE6/TMPRSS2 cells were seeded to 24-well plates (2 × 10^5^ cells/well) 1 day before the experiment. The medium was removed, and 100 μL of the samples were added to each well. After 30 min, the samples were removed, and the cells were washed three times with serum-free MEM. The cells were then cultured in 1 mL of DMEM containing 2% fetal bovine serum, 200 units/mL of penicillin, 200 μg/mL of streptomycin, and 0.25 μg/mL of amphotericin B in an atmosphere with 5% CO_2_ and at 37 °C for up to 5 days. Cytopathic effects (CPEs) were macroscopically observed each day. When CPEs appeared, the culture supernatant was collected (passage #0). The isolated viruses were passaged once (passage #1) and titrated using focus-forming assays.

#### Viral sequencing and phylogenetic analysis

For viral sequencing, RNA was extracted from the culture supernatant of the isolated viruses (passage #0) using the Qiagen Viral RNA Mini kit (QIAGEN). cDNA synthesis was performed using the GenNext RamDA-seq Single Cell Kit (TOYOBO). An Illumina library was constructed using the Nextera XT DNA Library Prep Kit (Illumina) and Nextera XT Index Kit v2 (Illumina). The resulting DNA libraries were sequenced using the NovaSeq6000 instrument (Illumina). The obtained reads were analyzed using Cutadapt to trim adapters; bwa to map the reads to the reference genome (NC_045512.2); Mutect2 to call mutations; snpEff to make annotations; and HaplotypeCaller and bcftools to make consensus sequences.

A phylogenetic tree was inferred from the alignment using the maximum likelihood approach in W-IQ-TREE.^51^ The best-fit model was selected by ModelFinder,^52^ and the ultrafast bootstrap^53^ with 1,000 replicates was calculated. The viruses used for the phylogenetic analyses have been listed in Table S3. We used BLAST to obtain similar sequences to the virus from the patient using the NCBI database. We picked 17 sequences based on the highest identity score. As references, we added 8 sequences from various lineages. We then added 4 sequences from the same hospital and 16 sequences from the same prefecture of Japan, all of which were isolated and analyzed in-house during the period in which the patient was infected and recovered after 240 days of being infected.

#### *In vitro*T-cell expansion and stimulation

Cryopreserved peripheral blood mononuclear cells (PBMCs) were thawed and washed with RPMI 1640 medium (Sigma-Aldrich) supplemented with 5% human AB serum (Gemini Bio), penicillin (Sigma-Aldrich), streptomycin (MP Biomedicals), and 2-mercaptoethanol (Nacalai Tesque). The cells were expanded with 4 μg/mL of single peptides derived from the SARS-CoV-2 Wuhan-Hu-1 strain in the same medium for 12 to 15 days. We used 15-mer peptides since they are widely used for activating both CD4^+^ T cells and CD8^+^ T cells,^57^ presumably because the exo-degradation of peptides during culture creates the appropriate length of peptides for MHC class I binding. Recombinant human IL-2 (10 IU/mL; Peprotech), human IL-7 (5 ng/mL; BioLegend), and human IL-15 (5 ng/mL; Peprotech) were supplied every 3 days from day 2 onwards. For the re-stimulation assay, the PBMCs that had been expanded *in vitro* were harvested at the indicated time points and washed before being re-stimulated with 4 μg/mL of the Wuhan or mutant peptides for the indicated durations. The resultant cells were used for subsequent assays. Cytokines were analyzed using the LEGENDplex Human CD8/NK Panel (13-plex) (BioLegend). An ELISPOT assay was performed using a human interferon-γ/IL-4 Double-Color ELISPOT (ImmunoSpot). The PBMCs were stimulated with peptide pools derived from proteins of the SARS-CoV-2 Wuhan-Hu-1 strain (1 μg/mL per peptide; JPT) for 48 h. The expression of the cytokines was determined based on the manufacturer’s protocol. The single peptides used for the stimulation of the T cells have been listed in Table S6.

#### Reporter cell establishment and stimulation

TCR-α and-β chain cDNA sequences were introduced into a mouse T-cell hybridoma with an NFAT-GFP reporter gene^44^ using retroviral vectors. TCR-reconstituted cells were co-cultured with 1 μg/mL of single peptides in the presence of PBMCs from the patient as antigen-presenting cells. After 20 h, cell activation was assessed based on the expression of GFP and CD69.

#### Single-cell-based transcriptome and TCR repertoire analyses

Single-cell libraries were prepared using reagents from 10× Genomics following the manufacturer’s instructions. RNA from the cells was reverse-transcribed; cDNA was amplified for 14 cycles; and up to 50 ng of cDNA were used for the gene expression and TCR libraries. Clustering was performed based on the top-4,000 highly variable genes, and the cell types were annotated as previously described.^58^

The single-cell RNA-seq fastq files were processed using 10× Genomics Cell Ranger version 6.0^59^ with default settings to yield the gene expression profiles of the hashtag-attached cell barcodes. The transcriptomes of the 13,777 and 13,078 barcodes from two different libraries were merged and subjected to downstream analyses using Scanpy 1.8.1.^60^ Information on the T-cell clonotypes was integrated with the gene expression profiles of the single-cell RNA-seq data using Scirpy.^61^ The read counts of the hashtags of the cell barcodes were extracted and scaled from 0 to 10. The samples from which the cell barcodes originated were then estimated based on the hashtag read counts of each barcode. To avoid the potential bias of clustered results, ribosomal genes, *TRAV*, *TRAJ*, *TRBJ*, *TRBV*, *IGHV*, *IGKV*, and *IGLV* were excluded from the downstream analyses. Scrublet^62^ was used to predict and filter potential doublets. The distributions of the read counts, gene counts per barcode, ribosomal gene concentration, mitochondrial gene concentration, and hemoglobin gene concentration within the samples were each fitted with a mixture of two Gaussian distributions.^63^ Filtration thresholds for the quality control of the cell barcodes were then determined based on the distance of the fitted mean values of the Gaussian distributions. The resulting 25,961 cells, which consisted of 9,708 cells from the M121 sample, 7,669 cells from the S1145 sample, 7,082 cells from the 1ab2973 sample, and 1,502 cells from the 3a25 sample, were used in the final analysis (see below for each sample name). The top-4,000 highly variable genes were selected using Cellranger flavor integrated with Scanpy.^59^ Batch effect correction was conducted using BBKNN.^64^ Leiden clustering^65^ and PAGA graphs^66^ with Reingold–Tilford layouts^67^ were integrated with UMAP.^68^ The cell typing of the T cells was conducted by mapping the transcriptomic data onto the reference PBMC dataset published by Hao et al.,^58^which was downloaded from the UCSC Cell Browser Homepage.^69^

#### Bulk TCR sequencing and analysis

A total of 3 x 10^5^ fresh PBMCs were lysed in 200 μL of QIAzol (QIAGEN). Full-length cDNA was synthesized using SMARTer (Takara Bio) before variable regions of the *TCR-α* and*-β* genes were amplified. Each pair of reads was assigned a clonotype (defined as *TR(A/B)V* and *TR(A/B)J* genes and the complementarity-determining region 3) using MiXCR software.^70^

#### Monocyte-derived dendritic cells

CD14^+^ cells were isolated from PBMCs using human CD14 MicroBeads (Miltenyi Biotec) and cultured in RPMI 1640 medium supplemented with 5% human AB serum, 0.1 mM Non-Essential Amino Acid Solution (Gibco-BRL), 1 mM sodium pyruvate (Gibco-BRL), 10 ng/mL of human granulocyte–macrophage colony-stimulating factor (GM-CSF) (Peprotech), and 10 ng/mL of IL-4 (Peprotech) for 13 days.

### Quantification and statistical analysis

The cytokines were measured in duplicate. The data are shown as mean ± standard deviation.

## Data Availability

• Single-cell TCR- and RNA-sequencing data have been deposited in Gene Expression Omnibus (GEO) datasets (accession number: GSE190895). The newly obtained viral sequence data may be downloaded from GISAID (https://www.gisaid.org) as accession numbers listed in key resources table. Patient-related data not included in the paper were generated as part of the clinical examinations and may be subjected to patient confidentiality. • This paper does not report original code. • Any additional information required to reanalyze the data reported in this paper is available from the lead contact upon request.
