# Supplementary material for: COVID-19 relapse associated with SARS-CoV-2 evasion from CD4+ T-cell recognition in an agammaglobulinemia patient
Source: iScience. 2023 Apr 20;26(5):106685. doi: 10.1016/j.isci.2023.106685 (PMC10116114; doi:10.1016/j.isci.2023.106685)
Supplement: Document S1. Figures S1 and S2 and Tables S1–S9 [file mmc1.pdf]

**iScience, Volume *26***

## Supplemental information

# COVID-19 relapse associated with SARS-CoV-2 evasion from CD4$^{+}$ T-cell recognition in an agammaglobulinemia patient

**Ryo Morita, Ritsuko Kubota-Koketsu, Xiuyuan Lu, Tadahiro Sasaki, Emi E. Nakayama, Yu-chen Liu, Daisuke Okuzaki, Daisuke Motooka, James Badger Wing, Yasunori Fujikawa, Yuji Ichida, Kiyoko Amo, Tetsushi Goto, Junichi Hara, Michinori Shirano, Sho Yamasaki, and Tatsuo Shioda**

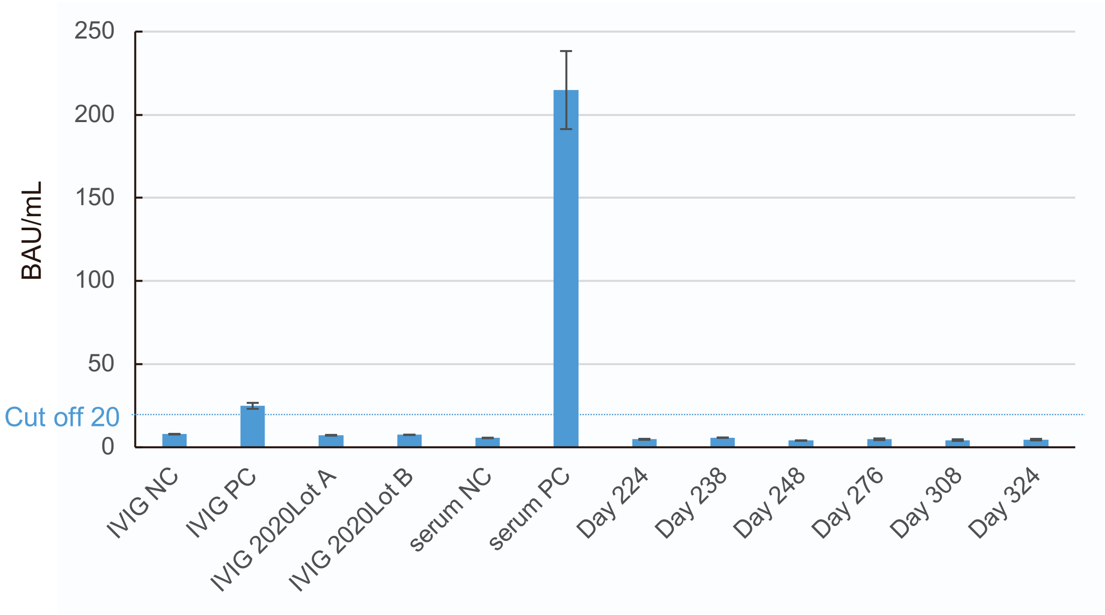


**Figure S1. The anti-SARS-CoV-2 spike (S) protein in the patient plasma (related to Figure 1).** Binding antibody titers against the SARS-CoV-2 S protein in the patient plasma and intravenous immunoglobulin (IVIG) lots administered to the patient during the study. BAU, binding antibody unit; NC, negative control; PC, positive control. Values over 20 BAU/mL were considered positive. Error bars indicate mean ± SD of triplicate measurements.

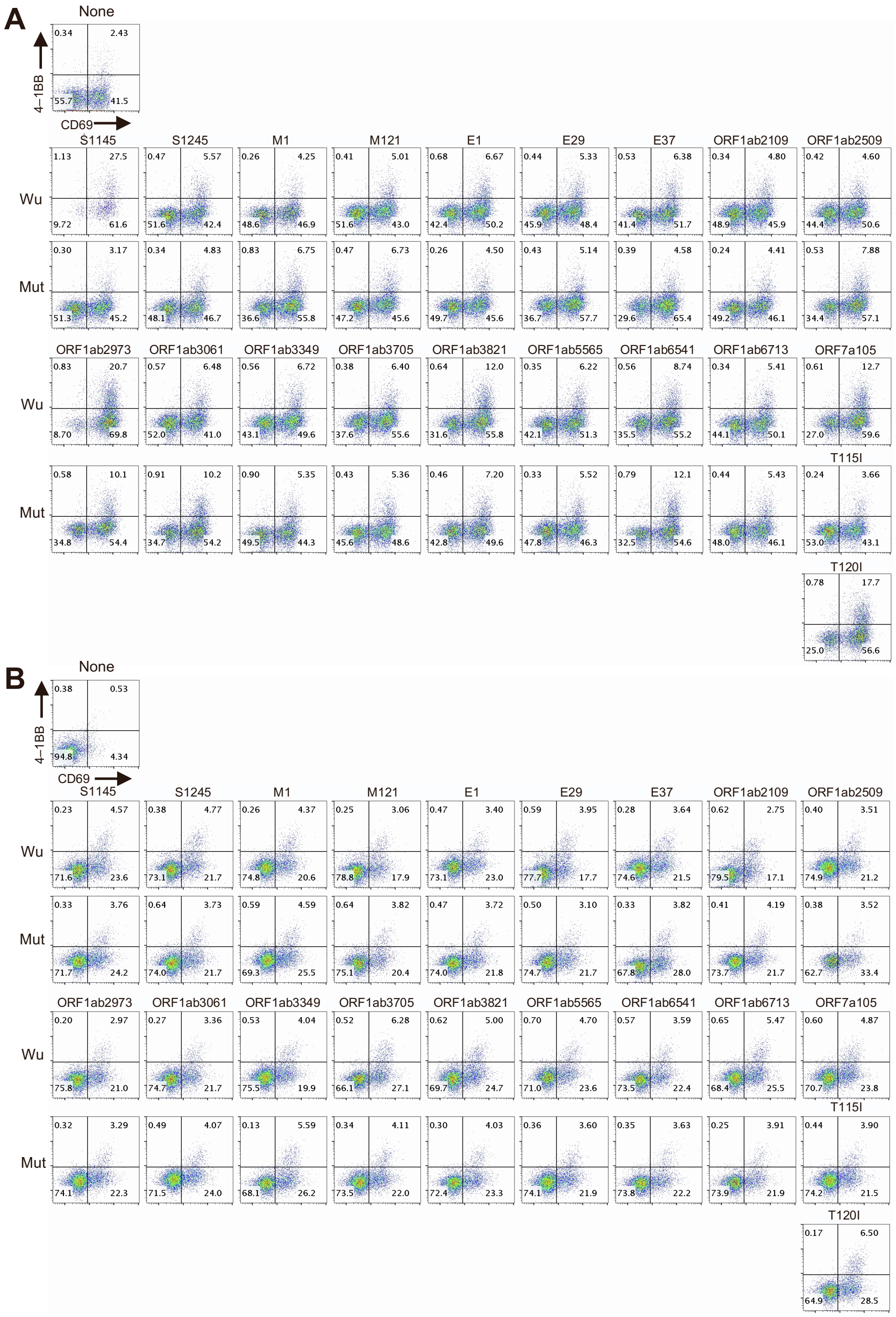


**Figure S2. Responses of CD4+ and CD8+ T cells to the Wuhan and mutant peptides of SARS-CoV-2 (related to Figure 3).**

PBMCs collected from the donor on day 308 were stimulated with 19 pooled Wuhan peptides that covered the mutations detected in the donor for 13 days. CD4+ and CD8+ T cells were then sorted separately and re-stimulated with nothing (None), individual Wuhan peptides (Wu), or corresponding mutant peptides (Mut) in the presence of autologous monocyte-derived dendritic cells for 20 h. CD69+4-1BB+ populations within the CD4+ T-cell gate (A) and CD69+4-1BB+ populations within the CD8+ T-cell gate (B) are shown.

**Table S1. HLA type, lymphocyte subsets, and activity of neutrophils and natural killer cells in the patient (related to Figure 1 and Figure 5).**

| Lymphocyte subset | % lymphocytes | Activity of neutrophils, and natural killer cells | % | |
|---|---|---|---|---|
| | Day 276 | | Day 276 | Day 790 |
| **CD3** | 92.6 | **Neutrophil phagocytosis function** | 44.9 | 40.8 |
| **CD4** | 39.2 | | | |
| **CD8** | 46.1 | **Neutrophil sterilizing function** | 99.8 | 99.9 |
| **CD13** | 0.5 | | | |
| **CD19** | 0.0 | **Natural killer cell activity** | 33.1 | 47.4 |

| HLA type | Allele 1 | Allele 2 |
|---|---|---|
| **A** | 11:01:01 | 31:01:02 |
| **B** | 15:01:01 | 48:01:01 |
| **C** | 04:01:01 | – |
| **DRB1** | 04:06:01 | 09:01:02 |
| **DRB345** | 01:03:01 | – |
| **DQA1** | 03:01:01 | – |
| **DQB1** | 03:02:01 | 03:03:02 |
| **DPA1** | 02:02:02 | – |
| **DPB1** | 05:01:01 | – |

**Table S2. Results of the multiplex PCR testing of sputum samples for 19 respiratory tract infections (BioFire® FilmArray Respiratory Panel 2.1) (related to Figure 1).**

| Viruses and Bacteria | Result |
|---|---|
| **Adenovirus** | not detected |
| **Coronavirus 229E** | not detected |
| **Coronavirus HKU1** | not detected |
| **Coronavirus NL63** | not detected |
| **Coronavirus OC43** | not detected |
| **SARS-CoV-2** | detected |
| **Human Metapneumovirus** | not detected |
| **Human Rhinovirus/Enterovirus** | not detected |
| **Influenza A** | not detected |
| **Influenza B** | not detected |
| **Parainfluenza Virus 1** | not detected |
| **Parainfluenza Virus 2** | not detected |
| **Parainfluenza Virus 3** | not detected |
| **Parainfluenza Virus 4** | not detected |
| **Respiratory Syncytial Virus** | not detected |
| ***Bordetella parapertussis* (IS*1001*)** | not detected |
| ***Bordetella pertussis* (*ptxP*)** | not detected |
| ***Chlamydia pneumoniae*** | not detected |
| ***Mycoplasma pneumoniae*** | not detected |

**Table S3. Data from the blood test results obtained during the observation period, particularly data related to the severity of COVID–19 (related to Figure 1).**

| Days since first PCR(+) | 0 | 1 | 3 | 7 | 21 | 35 | 63 | 92 | 140 | 175 | 181 | 209 | 216 | 224 | 230 | 235 |
|---|---|---|---|---|---|---|---|---|---|---|---|---|---|---|---|---|
| WBC(/μL) | 8,680 | 8,620 | 6,830 | 6,790 | 9,050 | 8,580 | 7,360 | 10,740 | 9,420 | 9,340 | 8,850 | 9,930 | 9,530 | 8,090 | 9,090 | 8,170 |
| Lymph (/μL) | 930 | 1,030 | 1,340 | 2,170 | 1,340 | 1,170 | 1,320 | 1,180 | 890 | 650 | 1,120 | 960 | 1,040 | 1,150 | 1,140 | 1,450 |
| Hb (g/dL) | 14.7 | 14.0 | 14.3 | 15.5 | 14.9 | 13.9 | 13.9 | 13.9 | 13.0 | 12.5 | 12.9 | 13.4 | 13.4 | 13.2 | 12.6 | 12.4 |
| Plt (/μL) | 127,000 | 151,000 | 170,000 | 198,000 | 130,000 | 131,000 | 128,000 | 165,000 | 197,000 | 171,000 | 185,000 | 219,000 | 195,000 | 173,000 | 208,000 | 154,000 |
| Alb (g/dL) | .. | 3.6 | 3.5 | 4.0 | 4.1 | 3·5 | .. | 3.8 | .. | 3.4 | 3.7 | 3.9 | 3.9 | 3.9 | 3.7 | 3.4 |
| ALT (IU/L) | 20 | 17 | 14 | 27 | 22 | 25 | 25 | 35 | 18 | 13 | 14 | 16 | 18 | 14 | 15 | 11 |
| LDH (IU/L) | 157 | 155 | 126 | 138 | 149 | 145 | 147 | 152 | 177 | 138 | 137 | 145 | 159 | 160 | 159 | 149 |
| CK (IU/L) | 30 | 32 | 23 | 24 | 30 | 25 | 28 | 24 | 21 | 19 | 20 | 22 | 22 | 23 | 23 | 18 |
| Cre (mg/dL) | 0.55 | 0.56 | 0.58 | 0.65 | 0.58 | 0.61 | 0.62 | 0.49 | 0.47 | 0.42 | 0.47 | 0.50 | 0.48 | 0.50 | 0.52 | 0.54 |
| Troponin (pg/mL) | .. | .. | .. | .. | .. | .. | .. | .. | .. | .. | .. | .. | .. | <0.003 | .. | .. |
| PT (s) | .. | 11.2 | 11.4 | 11.1 | 11.4 | 10.9 | .. | .. | .. | 12.1 | .. | .. | 11.6 | 11.4 | .. | 12.0 |
| D-dimer (μg/mL) | .. | 0.6 | 0.6 | 0.5 | 0.5 | .. | .. | .. | .. | .. | .. | .. | .. | <0.5 | .. | .. |
| CRP (mg/dL) | 2.37 | 1.95 | 1.77 | 1.55 | 2.71 | 3.06 | 4.15 | 3.61 | 3.38 | 5.06 | 3.69 | 3.67 | 4.85 | 5.08 | 4.73 | 7.01 |
| Ferritin (μg/L) | 135 | 251 | 258 | 247 | 141 | 319 | 346 | | 254 | 221 | 208 | 183 | 164 | 163 | 156 | .. |
| IL-6 (pg/mL) | .. | .. | .. | .. | .. | .. | .. | .. | .. | .. | .. | 21.9 | 21.5 | 18.9 | 17.3 | .. |
| Procalcitonin (ng/mL | .. | 0.06 | .. | .. | .. | .. | .. | .. | .. | 0.06 | .. | .. | .. | 0.03 | .. | 0.06 |
| KL-6 (IU/mL) | .. | 185 | 188 | 225 | 236 | .. | .. | .. | .. | 430 | .. | .. | .. | 522 | .. | .. |
| IgG (mg/dL)* | 964 | 2,183 | .. | .. | 1,137 | 1,349 | 1,192 | .. | 954 | 1,695 | 1,425 | 1,478 | 1,248 | 978 | 1,899 | .. |

| Days since first PCR(+) | 237 | 238 | 239 | 240 | 241 | 242 | 243 | 244 | 246 | 248 | 252 | 258 | 265 | 276 | 308 | 336 |
|---|---|---|---|---|---|---|---|---|---|---|---|---|---|---|---|---|
| WBC(/μL) | 8,720 | 7,400 | 6,420 | 3,940 | 2,860 | 3,800 | 5,010 | 5,430 | 6,670 | 6,560 | 13,580 | 9,300 | 7,680 | 9,180 | 5,650 | 4,990 |
| Lymph (/μL) | 920 | 1,030 | 920 | 1,080 | 1,290 | 1,240 | 1,060 | 1,860 | 2,160 | 1,770 | 2,340 | 1,570 | 1,660 | 1,520 | 1,040 | 1,220 |
| Hb (g/dL) | 12.2 | 12.0 | 11.6 | 11.2 | 11.6 | 11.3 | 11.2 | 11.5 | 12.0 | 12.4 | 12.0 | 11.5 | 11.5 | 10.9 | 10.6 | 10.4 |
| Plt (/μL) | 151,000 | 147,000 | 157,000 | 162,000 | 144,000 | 161,000 | 168,000 | 175,000 | 204,000 | 209,000 | 211,000 | 152,000 | 145,000 | 185,000 | 134,000 | 134,000 |
| Alb (g/dL) | 3.5 | 3.6 | 3.4 | 3.3 | 3.4 | 3.3 | 3.2 | 3.4 | 3.5 | 3.8 | 3.9 | 4.1 | 4.4 | 4.5 | 4.6 | 4.8 |
| ALT (IU/L) | 11 | 12 | 14 | 13 | 14 | 25 | 47 | 50 | 62 | 63 | 39 | 52 | 35 | 27 | 17 | 17 |
| LDH (IU/L) | 157 | 164 | 179 | 164 | 158 | 167 | 179 | 162 | 166 | 161 | 233 | 151 | 156 | 164 | 141 | 163 |
| CK (IU/L) | 20 | 19 | 17 | 18 | 18 | 18 | 22 | 23 | 24 | 24 | 41 | 50 | 95 | 79 | 56 | 70 |
| Cre (mg/dL) | 0.51 | 0.48 | 0.49 | 0.48 | 0.48 | 0.44 | 0.49 | 0.44 | 0.48 | 0.53 | 0.60 | 0.59 | 0.55 | 0.54 | 0.68 | 0.69 |
| Troponin (pg/mL) | .. | 0.003 | .. | .. | .. | .. | .. | .. | 0.003 | .. | .. | .. | .. | .. | .. | .. |
| PT (s) | .. | 11.7 | 11.6 | 11.9 | 12.1 | 12.5 | 12.5 | 12.5 | 12.4 | 12.2 | 11.3 | 11.0 | 11.2 | 11.3 | 11.7 | 11.3 |
| D-dimer (μg/mL) | .. | 0.5 | 0.5 | <0.5 | 0.5 | 0.5 | <0.5 | <0.5 | <0.5 | <0.5 | <0.5 | <0.5 | <0.5 | <0.5 | <0.5 | |
| CRP (mg/dL) | 7.49 | 7.01 | 7.25 | 5.88 | 2.70 | 1.29 | 0.64 | 0.48 | 0.25 | 0.33 | 0.34 | 0.27 | 0.13 | 0.16 | 0.06 | 0.09 |
| Ferritin (μg/L) | 193 | 207 | 204 | 208 | 237 | 212 | 191 | 169 | 133 | 96 | 57 | 32 | 17 | 11 | 6 | 9 |
| IL-6 (pg/mL) | .. | 20.8 | 31.5 | 9.1 | 4.9 | 6.2 | 6.0 | 2.0 | 3.9 | 3.3 | 4.1 | 3.8 | 1.8 | 2.3 | <1.5 | 1.9 |
| Procalcitonin (ng/mL | .. | 0.05 | .. | .. | .. | .. | .. | .. | 0.05 | .. | .. | .. | .. | 0.06 | .. | .. |
| KL-6 (IU/mL) | .. | 510 | 476 | 480 | 516 | 508 | 516 | 550 | 567 | 594 | 494 | 398 | 358 | 266 | 170 | 156 |
| IgG (mg/dL)[a] | 1,371 | 1,372 | 1,229 | 1,199 | 1,227 | 1,141 | 1,148 | 1,131 | 1,108 | 1,134 | 1,021 | 1,909 | 1,557 | 1,146 | 1,150 | 1,197 |

The green shaded area indicates remdesivir administration.

[a]The patient received regular intravenous immunoglobulin transfusions.

**Table S4. Detection of infectious viruses and viral RNA in clinical specimens (related to Figure 2).**

| Sample | Collection day | Virus titers (FFUs/mL) | Virus isolation | qRT–PCR (Ct value) | Viral RNA (copies/μL) |
|---|---|---|---|---|---|
| **Saliva** | Day 0 | not done | not done | 31.9 | 2.6E+01 |
| **Nasopharyngeal swab** | Day 1 | not done | not done | 21.3 | 1.1E+05 |
| **Nasopharyngeal swab**[a] | Day 1 | 2,160 | + | 23.0 | 4.4E+04 |
| **Saliva** | Day 63 | not done | not done | 34.7 | 3.3E+00 |
| **Saliva** | Day 77 | not done | not done | 33.8 | 6.4E+00 |
| **Saliva** | Day 77 | <5 | – | – | NA[b] |
| **Saliva** | Day 92 | not done | not done | – | NA |
| **Saliva** | Day 92 | not done | not done | – | NA |
| **Sputum** | Day 175 | not done | not done | 35.9 | 1.0E+01 |
| **Sputum** | Day 175 | not done | – | 38.8 | 6.5E–01 |
| **Sputum** | Day 176 | not done | not done | 36.5 | 8.7E+00 |
| **Sputum** | Day 176 | not done | – | 37.0 | 2.2E+00 |
| **Sputum** | Day 181 | not done | not done | 35.3 | 3.9E+01 |
| **Sputum** | Day 196 | <2.5 | – | 37.6 | 1.8E+00 |
| **Sputum** | Day 204 | <2.5 | – | not done | NA |
| **Sputum** | Day 209 | <2.5 | – | 31.2 | 1.1E+00 |
| **Bronchoalveolar lavage fluid–1** | Day 218 | 10 | + | – | NA |
| **Bronchoalveolar lavage fluid–2** | Day 218 | 15 | + | 31.5 | 9.1E+01 |
| **Bronchoalveolar lavage fluid–3**[a] | Day 218 | 17.5 | + | 32.1 | 6.2E+01 |
| **Lung tissue (by biopsy)**[a] | Day 218 | 15 | + | not done | NA |
| **Suction phlegm** | Day 218 | <2.5 | – | 35.6 | 6.5E+00 |
| **Sputum**[a] | Day 224 | 5 | + | 35.0 | 9.6E+00 |
| **Saliva** | Day 224 | <5 | – | 39.8 | 4.3E–01 |
| **Nasopharyngeal swab** | Day 237 | not done | – | – | NA |
| **Sputum** | Day 237 | not done | – | 37.0 | 3.0E+00 |
| **Sputum** | Day 238 | not done | – | 34.4 | 1.8E+01 |
| **Urine** | Day 238 | not done | – | – | NA |
| **Whole blood** | Day 238 | not done | – | – | NA |
| **Sputum–1** | Day 239 | 10 | + | 31.0 | 1.9E+02 |
| **Sputum–2**[a] | Day 239 | 130 | + | 33.4 | 3.6E+01 |
| **Sputum** | Day 240 | <5 | – | – | NA |
| **Sputum** | Day 241 | <5 | – | 43.7 | 2.9E–02 |
| **Sputum–1** | Day 242 | <5 | – | 42.0 | 9.5E–02 |
| **Sputum–2** | Day 242 | <5 | – | – | NA |
| **Sputum** | Day 243 | <5 | – | – | NA |
| **Sputum** | Day 244 | <5 | – | – | NA |
| **Sputum** | Day 245 | <5 | – | – | NA |
| **Sputum** | Day 246 | <5 | – | – | NA |
| **Sputum** | Day 247 | <5 | – | – | NA |
| **Sputum** | Day 248 | <5 | – | – | NA |
| **Sputum** | Day 251 | <5 | – | – | NA |
| **Sputum** | Day 252 | <5 | – | – | NA |
| **Sputum** | Day 253 | <5 | – | – | NA |
| **Sputum** | Day 255 | <5 | – | – | NA |
| **Nasopharyngeal swab** | Day 255 | <5 | – | – | NA |
| **Sputum** | Day 258 | <5 | – | – | NA |
| **Nasopharyngeal swab** | Day 258 | <5 | – | – | NA |
| **Sputum** | Day 260 | <5 | – | – | NA |
| **Sputum** | Day 262 | <5 | – | – | NA |
| **Sputum** | Day 265 | <5 | – | – | NA |
| **Sputum** | Day 267 | <5 | – | – | NA |
| **Sputum** | Day 269 | <5 | – | – | NA |
| **Sputum** | Day 273 | <5 | – | – | NA |
| **Sputum** | Day 276 | <5 | – | – | NA |
| **Sputum** | Day 308 | not done | – | – | NA |

The underlined samples were stored at –80°C and tested retrospectively.
[a]Samples tested for the viral genome analysis.
[b]Not applicable.

**Table S5. List of intravenous immunoglobulin (IVIG) products administered to the patient (related to Figure 1).**

| Day | Product | Company | Period of blood collection | Dosage (g) |
|---|---|---|---|---|
| -112 | GAMMAGARD Solvent/Detergent Treated Intravenous 5g | Baxalta US Inc. | –2019 May | 40 |
| | Venoglobulin IH 10％ I.V.10g/100mL | Japan Blood Products Organization | 2017 Oct | 10 |
| -84 | GAMMAGARD Solvent/Detergent Treated Intravenous 5g | Baxalta US Inc. | –2019 May | 40 |
| | Venoglobulin IH 10％ I.V.10g/100mL | Japan Blood Products Organization | 2018 Jan | 10 |
| -56 | GAMMAGARD Solvent/Detergent Treated Intravenous 5g | Baxalta US Inc. | –2019 Aug | 40 |
| | Venoglobulin IH 10％ I.V.10g/100mL | Japan Blood Products Organization | 2018 Jan | 10 |
| -28 | GAMMAGARD Solvent/Detergent Treated Intravenous 5g | Baxalta US Inc. | –2019 Aug | 40 |
| | Venoglobulin IH 10％ I.V.10g/100mL | Japan Blood Products Organization | 2018 Jan | 10 |
| 0 | GAMMAGARD Solvent/Detergent Treated Intravenous 5g | Baxalta US Inc. | –2019 Aug | 40 |
| | Venoglobulin IH 10％ I.V.10g/100mL | Japan Blood Products Organization | 2018 Jan | 10 |
| 21 | GAMMAGARD Solvent/Detergent Treated Intravenous 5g | Baxalta US Inc. | –2019 Aug | 50 |
| | Venoglobulin IH 10％ I.V.10g/100mL | Japan Blood Products Organization | 2018 Jun | 10 |
| 42 | GAMMAGARD Solvent/Detergent Treated Intravenous 5g | Baxalta US Inc. | –2019 Aug | 50 |
| | Venoglobulin IH 10％ I.V.10g/100mL | Japan Blood Products Organization | 2018 Jun | 10 |
| 63 | GAMMAGARD Solvent/Detergent Treated Intravenous 5g | Baxalta US Inc. | –2019 Aug | 50 |
| | Venoglobulin IH 10％ I.V.10g/100mL | Japan Blood Products Organization | 2018 Jun | 10 |
| | Venoglobulin IH 10% I.V.5g/50mL | Japan Blood Products Organization | 2018 Aug | 10 |
| 84 | Privigen 10％ I.V.Drip Infusion 10g/100mL | CSL Behring | 2018 Apr–2019 Mar | 60 |
| 112 | Privigen 10％ I.V.Drip Infusion 10g/100mL | CSL Behring | 2018 Sep–2019 May[a] | 70 |
| 140 | Privigen 10％ I.V.Drip Infusion 10g/100mL | CSL Behring | 2018 Sep–2019 May[a] | 70 |
| 168 | Privigen 10％ I.V.Drip Infusion 10g/100mL | CSL Behring | 2018 Sep–2019 May[a] | 70 |
| 196 | Privigen 10％ I.V.Drip Infusion 10g/100mL | CSL Behring | 2018 Sep–2019 May[a] | 70 |
| 224 | Privigen 10％ I.V.Drip Infusion 10g/100mL | CSL Behring | 2018 Sep–2019 May[a] | 70 |
| 262 | Privigen 10％ I.V.Drip Infusion 10g/100mL | CSL Behring | 2018 Sep–2019 May[a] | 70 |
| 280 | Privigen 10％ I.V.Drip Infusion 10g/100mL | CSL Behring | 2018 Sep–2019 May[a] | 70 |
| 308 | Privigen 10％ I.V.Drip Infusion 10g/100mL | CSL Behring | 2018 Sep–2019 May[a] | 70 |
| 336 | Privigen 10％ I.V.Drip Infusion 10g/100mL | CSL Behring | 2018 Sep–2019 May[a] | 70 |
| 364 | Privigen 10％ I.V.Drip Infusion 10g/100mL | CSL Behring | 2018 Sep–2019 May[a] | 70 |
| 392 | Privigen 10％ I.V.Drip Infusion 10g/100mL | CSL Behring | 2018 Sep–2019 May[a] | 70 |
| 420 | Privigen 10％ I.V.Drip Infusion 10g/100mL | CSL Behring | 2018 Sep–2019 May[a] | 70 |
| 448 | Privigen 10％ I.V.Drip Infusion 10g/100mL | CSL Behring | 2019 Apr–2019 Jul[b] | 70 |

[a]IVIG 2020Lot A was tested for anti-SARS-CoV-2 spike protein antibodies using ELISA (Figure S1).
[b]IVIG 2020Lot B was tested for anti-SARS-CoV-2 spike protein antibodies using ELISA (Figure S1).

**Table S6. List of viruses used in the phylogenetic analysis (related to Figure 2 and the STAR Methods).**

| | Accession ID | | Virus name | Pango lineage | Clade |
|---|---|---|---|---|---|
| | GISAD | NCBI | | | |
| **Day_218_BALF** | EPI_ISL_4935777 | .. | hCoV–19/Japan/RIMD–DVI–C10B/2021 | B.1.1.284 | GR |
| **Day_218_Lung** | EPI_ISL_4935949 | .. | hCoV–19/Japan/RIMD–DVI–C10L/2021 | B.1.1.284 | GR |
| **Day_224_Sputum** | EPI_ISL_4936095 | .. | hCoV–19/Japan/RIMD–DVI–C16/2021 | B.1.1.284 | GR |
| **Day_1_NPS** | EPI_ISL_4936243 | .. | hCoV–19/Japan/RIMD–DVI–H06/2020 | B.1.1.284 | GR |
| **Day_239_Sputum** | EPI_ISL_4936533 | .. | hCoV–19/Japan/RIMD–DVI–C31/2021 | B.1.1.284 | GR |
| **OCGH_P2_2021** | EPI_ISL_4929525 | .. | hCoV–19/Japan/RIMD–DVI–C26H/2021 | R.1 | .. |
| **OCGH_P3_2021** | EPI_ISL_4930839 | .. | hCoV–19/Japan/RIMD–DVI–C26W/2021 | R.1 | .. |
| **OCGH_P4_2021** | EPI_ISL_4931056 | .. | hCoV–19/Japan/RIMD–DVI–D09/2021 | B.1.1.7 | GRY |
| **OCGH_P6_2021** | EPI_ISL_4931846 | .. | hCoV–19/Japan/RIMD–DVI–D21/2021 | B.1.1.7 | GRY |
| **OIPH_1_2020** | EPI_ISL_4932136 | .. | hCoV–19/Japan/OIPH14/2020 | A | S |
| **OIPH_2_2020** | EPI_ISL_4932550 | .. | hCoV–19/Japan/OIPH16/2020 | B.1.1.284 | GR |
| **OIPH_3_2020** | EPI_ISL_4932712 | .. | hCoV–19/Japan/OIPH21/2020 | B.1.1.284 | GR |
| **OIPH_24_2020** | EPI_ISL_4932860 | .. | hCoV–19Japan/OIPH29/2020 | B.1.1.284 | GR |
| **OIPH_35_2020** | EPI_ISL_4933015 | .. | hCoV–19/Japan/OIPH34/2020 | B.1.1.284 | GR |
| **OIPH_43_2020** | EPI_ISL_4933271 | .. | hCoV–19/Japan/OIPH6/2020 | B.1.1.285 | GR |
| **OIPH_71_2020** | EPI_ISL_4933542 | .. | hCoV–19/Japan/OIPH51/2020 | B.1.1.284 | GR |
| **OIPH_76_2020** | EPI_ISL_4933741 | .. | hCoV–19/Japan/OIPH54/2020 | B.1.1.284 | GR |
| **OIPH_92_2020** | EPI_ISL_4933859 | .. | hCoV–19Japan/OIPH61/2020 | B.1.1.284 | GR |
| **OIPH_123_2021** | EPI_ISL_4933990 | .. | hCoV–19/Japan/OIPH1/2021 | B.1.1.28 | .. |
| **OIPH11_2020** | EPI_ISL_4934247 | .. | hCoV–19/Japan/OIPH25/2020 | B.1.1.284 | GR |
| **OIPH19_2020** | EPI_ISL_4934751 | .. | hCoV–19/Japan/OIPH28/2020 | B.1.1.284 | GR |
| **OIPH127_2021** | EPI_ISL_4934873 | .. | hCoV–19/Japan/OIPH5/2021 | B.1.1.214 | GR |
| **OIPH128_2021** | EPI_ISL_4934996 | .. | hCoV–19/Japan/OIPH6/2021 | B.1.1.214 | GR |
| **OIPH133_2021** | EPI_ISL_4935185 | .. | hCoV–19J/apan/OIPH10/2021 | B.1.1.214 | GR |
| **OIPH135_2021** | EPI_ISL_4935429 | .. | hCoV–19/Japan/OIPH12/2021 | B.1.1.214 | GR |
| **Japan_TKYE626993_2020** | ..[a] | LC606017 | hCoV–19/Japan/TKYE626993/2020 | B.1.1.284 | GR |
| **Japan_TKYET29933_2020** | EPI_ISL_649137 | LC593812 | hCoV–19/Japan/TKYET29933/2020 | B.1.1.284 | GR |
| **Japan_TKYE618937_2020** | ..[a] | LC573279 | hCoV–19/Japan/TKYE618937/2020 | .. | .. |
| **Japan_TKYT22395_2020** | EPI_ISL_649132 | LC593807 | hCoV–19/Japan/TKYT22395/2020 | B.1.1.284 | GR |
| **Japan_TKYE630074_2020** | EPI_ISL_649138 | LC593813 | hCoV–19/Japan/TKYE630074/2020 | B.1.1.284 | GR |
| **Japan_TKYE624705_2020** | EPI_ISL_649135 | LC593810 | hCoV–19/Japan/TKYE624705/2020 | B.1.1.284 | GR |
| **Japan_TK–Y17873_2020** | EPI_ISL_486909 | LC567857 | hCoV–19/Japan/TK–Y17873/2020 | B.1.1.284 | GR |
| **Japan_TKYE618779_2020** | .. | LC573283 | hCoV–19/Japan/TKYE618779/2020 | .. | .. |
| **Japan_TKYE627000_2020** | EPI_ISL_649136 | LC593811 | hCoV–19/Japan/TKYE627000/2020 | B.1.1.284 | GR |
| **Japan_TKYE618749_2020** | .. | LC573282 | hCoV–19/Japan/TKYE618749/2020 | .. | .. |
| **Japan_TKYT22355_2020** | EPI_ISL_649127 | LC593802 | hCoV–19/Japan/TKYT22355/2020 | B.1.1.284 | GR |
| **Japan_TKYT22342_2020** | EPI_ISL_649126 | LC593801 | hCoV–19/Japan/TKYT22342/2020 | B.1.1.284 | GR |
| **Japan_TKYE618930_2020** | .. | LC573278 | hCoV–19/Japan/TKYE618930/2020 | .. | .. |
| **Japan_TKYE630094_2020** | EPI_ISL_649139 | LC593814 | hCoV–19/Japan/TKYE630094/2020 | B.1.1.284 | GR |
| **Japan_TKYT30095_2020** | EPI_ISL_649140 | LC593815 | hCoV–19/Japan/TKYT30095/2020 | B.1.1.284 | GR |
| **Japan_TKYE618928_2020** | .. | LC573276 | hCoV–19/Japan/TKYE618928/2020 | .. | .. |
| **Japan_TKYE618929_2020** | .. | LC573277 | hCoV–19/Japan/TKYE618929/2020 | .. | .. |
| **England_MILK–9E05B3_2020** | EPI_ISL_601443 | .. | hCoV–19/England/MILK–9E05B3/2020 | B.1.1.7 | GRY |
| **England_20168037604_2020** | EPI_ISL_466615 | .. | hCoV–19/England/20168037604/2020 | B.1.1 | GR |
| **Spain_CT–ISCIII–2013597_2020** | EPI_ISL_539548 | .. | hCoV–19/Spain/CT–ISCIII–2013597/2020 | B.1.177 | GV |
| **Canada_ON–PHL–8751_2020** | EPI_ISL_418345 | .. | hCoV–19/Canada/ON–PHL–8751/2020 | B.1 | GH |
| **Germany_BY–ChVir–929_2020** | EPI_ISL_406862 | .. | hCoV–19/Germany/BY–ChVir–929/2020 | B.1.153 | G |
| **Wuhan_WIV04_2019** | EPI_ISL_402124 | MN996528 | hCoV–19/Wuhan/WIV04/2019 | B | L |
| **Italy_LAZ–INMI–SPL1_2020** | EPI_ISL_412974 | .. | hCoV–19/Italy/LAZ–INMI–SPL1/2020 | B | V |
| **Guangdong_20SF012_2020** | EPI_ISL_403932 | .. | hCoV–19/Guangdong/20SF012/2020 | A | S |

[a]Not applicable.

**Table S7. SARS-CoV-2 mutations observed in the patient (related to Figure 2).**

| Nucleotide position | ORF | Day 1 reference | Allele | Amino acid substitution | Protein | Amino acid position in protein | Samples with mutation | Frequency in Cov–Glue data base[g] |
|---|---|---|---|---|---|---|---|---|
| **6617** | *ORF1ab* | G | T | G2118C | NSP3 | G1300C | A[b] | 102 |
| **7814** | *ORF1ab* | T | C | S2517P | NSP3 | S1699P | 224[c] | 80 |
| **9202** | *ORF1ab* | T | A | F2979L | NSP4 | F216L | 224 | 19 |
| **9316** | *ORF1ab* | T | C | V3017V | NSP4 | –[a] | A | |
| **9474** | *ORF1ab* | C | T | A3070V | NSP4 | A307V | A | 828 |
| **10323** | *ORF1ab* | A | G | K3353R | NSP5 | K90R | A | 19588 |
| **11266** | *ORF1ab* | G | A | L3667L | NSP6 | – | 224 | |
| **11388** | *ORF1ab* | T | C | V3708A | NSP6 | V139A | 239[d] | 4 |
| **11750** | *ORF1ab* | C | T | L3829F | NSP6 | L260F | A | 5,611 |
| **15654** | *ORF1ab* | C | T | D5130D | | – | 218L[e] | |
| **16993** | *ORF1ab* | T | C | Y5577H | NSP13 | Y253H | 224 | 545 |
| **19906** | *ORF1ab* | A | G | I6548V | NSP15 | I96V | 218B[f], 239 | 19 |
| **20421** | *ORF1ab* | T | G | D6719E | NSP15 | D267E | A | 148 |
| **21132** | *ORF1ab* | A | G | Q6956Q | | – | 218L | |
| **21637** | *S* | C | T | P25P | S | – | 218L | |
| **22987** | *S* | C | T | A475A | S | – | A | |
| **25008** | *S* | A | C | K1149T | S | K1149T | A | 0 |
| **25313** | *S* | G | A | G1251R | S | G1251R | 218B, 239 | 51 |
| **25487** | *ORF3a* | C | T | T32I | ORF3a | T32I | 239 | 1115 |
| **26270** | *E* | C | T | T9I | E | T9I | 239 | 1448 |
| **26333** | *E* | C | T | T30I | E | T30I | 218L | 298 |
| **26369** | *E* | A | G | Y42C | E | Y42C | A | 5 |
| **26550** | *M* | G | T | V10F | M | V10F | A | 9 |
| **26895** | *M* | C | T | H125Y | M | H125Y | A | 2030 |
| **27696** | *ORF7a* | T | C | F101F | ORF7a | – | 218B, 239 | |
| **27737** | *ORF7a* | C | T | T115I | ORF7a | T115I | A | 507 |
| **27752** | *ORF7a* | C | T | T120I | ORF7a | T120I | A | 2335 |

[a]Synonymous substitution.
[b]All four virus isolates.
[c]224, virus isolated from sputum on day 224.
[d]239, virus isolated from sputum on day 239.
[e]218L, virus isolated from lung tissue on day 218.
[f]218B, virus isolated from bronchoalveolar lavage fluid on day 218.
[g]Accessed on Sept 13, 2021. In the database, 867,980 sequences with D614G and 257,333 sequences with N501Y were found.

### Table S8. List of peptides used to stimulate T cells (related to Figure 3, 4, and 5).

| Peptide name | Sequence | |
|---|---|---|
| | Wuhan | Mutant |
| **E1** | MYSFVSEETGTLIVN | MYSFVSEEIGTLIVN |
| **E29** | VTLAILTALRLCAYC | VILAILTALRLCAYC |
| **E37** | LRLCAYCCNIVNVSL | LRLCACCCNIVNVSL |
| **M1** | MADSNGTITVEELKK | MADSNGTITFEELKK |
| **M121** | NVPLHGTILTRPLLE | NVPLYGTILTRPLLE |
| **ORF1ab 2109** | KPNELSRVLGLKTLA | KPNELSRVLCLKTLA |
| **ORF1ab 2509** | GQKTYERHSLSHFVN | GQKTYERHPLSHFVN |
| **ORF1ab 2973** | VRVVTTFDSEYCRHG | VRVVTTLDSEYCRHG |
| **ORF1ab 3061** | AYYFMRFRRAFGEYS | AYYFMRFRRVFGEYS |
| **ORF1ab 3349** | VLKLKVDTANPKTPK | VLKLRVDTANPKTPK |
| **ORF1ab 3705** | ARRVWTLMNVLTLVY | ARRAWTLMNVLTLVY |
| **ORF1ab 3821** | RYMNSQGLLPPKNSI | RYMNSQGLFPPKNSI |
| **ORF1ab 5565** | VPQEHYVRITGLYPT | VPQEHYVRITGLHPT |
| **ORF1ab 6541** | KRDAPAHISTIGVCS | KRDAPAHVSTIGVCS |
| **ORF1ab 6713** | SPFELEDFIPMDSTV | SPFELEEFIPMDSTV |
| **ORF3a 25** | PSDFVRATATIPIQA | PSDFVRAIATIPIQA |
| **ORF7a 105** | AAIVFITLCFTLKRKTE | AAIVFITLCFILKRKTE (T115I)<br>AAIVFITLCFTLKRKIE (T120I) |
| **S1145** | LDSFKEELDKYFKNH | LDSFTEELDKYFKNH |
| **S1245** | KGCCSCGSCCKFDED | KGCCSCRSCCKFDED |

Underlined amino acid sequences in the mutant peptides indicate the amino acids that differed from those in the Wuhan peptides.

**Table S9. V(D)J usages of clone-111 (related to Figure 4 and 5).**

| Clone ID | TRAV | CDR3a | TRAJ | TRBV | CDR3b | TRBJ |
|---|---|---|---|---|---|---|
| **111** | 13-2 | CAENPRFSGGYNKLIF | 4 | 6-1 | CASSGTGVDTQYF | 2-3 |
